# Multi-leveled Nanosilicate Implants Can Facilitate Near-Perfect Bone Healing

**DOI:** 10.1021/acsami.3c01717

**Published:** 2023-04-19

**Authors:** Mozhgan Keshavarz, Parvin Alizadeh, Firoz Babu Kadumudi, Gorka Orive, Akhilesh K. Gaharwar, Miguel Castilho, Nasim Golafshan, Alireza Dolatshahi-Pirouz

**Affiliations:** † Department of Materials Science and Engineering, Faculty of Engineering & Technology, Tarbiat Modares University, P.O. Box 14115-143, Tehran 14115-143, Iran; ‡ NanoBioCel Research Group, School of Pharmacy, University of the Basque Country (UPV/EHU), Vitoria-Gasteiz 01006, Spain; § DTU Health Tech, Center for Intestinal Absorption and Transport of Biopharmaceuticals, Technical University of Denmark, Kongens Lyngby 2800, Denmark; ∥ Biomedical Research Networking Centre in Bioengineering, Biomaterials and Nanomedicine (CIBER-BBN), Vitoria-Gasteiz 01006, Spain; ⊥ University Institute for Regenerative Medicine and Oral Implantology—UIRMI (UPV/EHU-Fundación Eduardo Anitua), Vitoria-Gasteiz 01006, Spain; # Bioaraba, NanoBioCel Research Group, Vitoria-Gasteiz 01006, Spain; ¶ Department of Biomedical Engineering, College of Engineering, Texas A&M University, College Station, Texas TX 77843, United States; ∇ Department of Biomedical Engineering, Eindhoven University of Technology, Eindhoven 5612 AE, The Netherlands; ○ Institute for Complex Molecular Systems, Eindhoven University of Technology, Eindhoven 5612 AE, The Netherlands; ⧫ Department of Orthopedics, University Medical Center Utrecht, Utrecht University, Utrecht 3508 GA, The Netherlands

**Keywords:** bio glass, alginate, laponite, hydrogels, mesenchymal stem cells, nanomaterials, nanosilicate

## Abstract

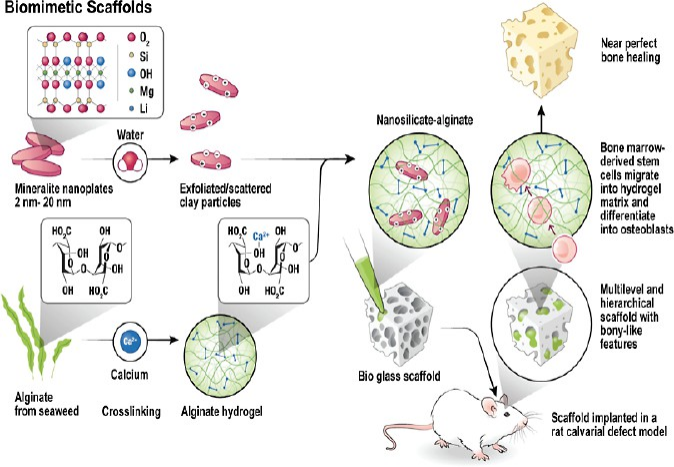

Several studies have shown that nanosilicate-reinforced scaffolds are suitable for bone regeneration. However, hydrogels are inherently too soft for load-bearing bone defects of critical sizes, and hard scaffolds typically do not provide a suitable three-dimensional (3D) microenvironment for cells to thrive, grow, and differentiate naturally. In this study, we bypass these long-standing challenges by fabricating a cell-free multi-level implant consisting of a porous and hard bone-like framework capable of providing load-bearing support and a softer native-like phase that has been reinforced with nanosilicates. The system was tested with rat bone marrow mesenchymal stem cells in vitro and as a cell-free system in a critical-sized rat bone defect. Overall, our combinatorial and multi-level implant design displayed remarkable osteoconductivity in vitro without differentiation factors, expressing significant levels of osteogenic markers compared to unmodified groups. Moreover, after 8 weeks of implantation, histological and immunohistochemical assays indicated that the cell-free scaffolds enhanced bone repair up to approximately 84% following a near-complete defect healing. Overall, our results suggest that the proposed nanosilicate bioceramic implant could herald a new age in the field of orthopedics.

## 1 Introduction

Loss of bone tissue can occur for a wide variety of reasons, including trauma, osteoporosis, and metastatic bone diseases.^1,2^ They currently affect 20 million people globally every year,^3^ making bone the second most common transplanted tissue in the world.^4^ Various techniques such as autografting, allografting, and xenografting have been employed for decades as the gold standard in the field;^5−8^ however, they still face some challenges such as infection and rejection risks that limit their clinical translation.^9−11^ A potential solution to overcome these limitations is bone tissue engineering. Here, a combination of scaffolds, hydrogels, and sometimes stem cells is used to generate bone tissue from scratch.^5,12^ However, despite the promise that the technique holds, it has still not unleashed its full potential. For example, the field still lacks biomaterial systems that fully mimic the delicate intricacies of native bone. Bone is essentially a multi-level organ composed of two different phases: a hard, inorganic phase and a soft, organic phase elegantly organized in a complex and hierarchical structure. The soft phase mainly consists of an extracellular matrix (ECM), constituted from nano- to micrometer-sized collagen fibers and various polysaccharides, while the hard phase is composed from an inorganic combinatorial mineral mixture consisting of hydroxyapatite, silica, magnesium, zinc, strontium, and lithium.^13^ For this reason, past and current developments have been focused on using such native-like materials for developing bone substitutes that can better resemble the native bone biological, chemical, and physical properties.^7,13^

Due to the high compatibility between bio glass (BG) ceramics and bone tissue, they have been used to reconstruct the hard phase of bone. BG are a class of ceramic materials with both a crystalline and glassy phase, in which their various components can be controlled to yield bioactive and biocompatible environments for bone tissue engineering.^14,15^ This is mainly related to the fact that they can quickly establish strong interfacial bonds with native bone through the formation of a native-like hydroxyapatite layer as a result of the dissolution of mineral products such as calcium (Ca), silica (Si), and phosphate (P).^16^ The released minerals in turn can enhance cell proliferation and differentiation and accelerate new bone formation.^15,17−23^ So, from both a chemical and biological point of view, BG exhibits many exciting properties suitable for bone healing.^5^ In particular, BG consisting of 60% SiO_2_–34% CaO–6% P_2_O_5_ (mol %) has shown a lot of promise in the field—mainly because it bonds more rapidly to the bone tissue than the other variants. This greatly reduces immune reactions and thus completely bypasses compromising fibrous tissue formations.^24−27^

On the organic side, alginate-based hydrogels present useful characteristics in the field such as ECM-like softness, biocompatibility, biodegradability, and low immune response.^28−33^ Their bioactivity can be improved via the incorporation of cell-adhesive RGD sequences or gelatin.^34−36^ However, RGD functionalization can sometimes be very tedious and gelatin quickly degrades in the body. Nanoscale biomaterials used in bone tissue engineering can be classified into one dimensional (1D), two dimensional (2D), and three dimensional (3D) based on their microstructural dimensions.^37^ Quantum dots, zero-dimensional nanomaterials composed of semiconductor nanocrystals ranging from 2 to 10 nm in size, possess advantageous properties of chemical and thermal stability, as well as optical properties. Furthermore, their unique optical properties allow quantum dots to be effectively utilized for bioimaging applications, cellular tracking, and in vivo and in vitro live cell imaging. Despite the potential biological and biomedical applications of quantum dots, their utilization has been limited by the presence of heavy toxic metals such as cadmium, lead, and mercury. Overall, quantum dots have not been widely employed in bone regeneration because of their small size and inadequate structural stability. Nanoflakes are 2D nanoparticles typically ranging from 10 to 100 nm and have a plate-like morphology with a substantial surface area. This feature renders them suitable for application such as coatings on implants for enhanced biocompatibility.^38−40^ Subsequently, 3D nanomaterials with distinct hierarchical structures are created from 1D and 2D nanoarchitectures, providing more advantageous characteristics for bone regeneration. Nanocrystals are 3D nanomaterials having a size range varying between 200 and 500 nm, which possess a high surface area, making them beneficial for bone development and integration, and thus, they are commonly applied as coatings to implants or incorporated as fillers for bone grafts to foster osseointegration. Owing to their properties, these nanomaterials differentiate in terms of their capacity to enhance cell attachment, proliferation, and bone restoration. The distinguishing characteristics between these nanomaterials are their physical properties, such as shape, particle dimension size, surface area, as well as composition. Overall, nanocrystals due to their higher surface area in comparison to quantum dots and nanoflakes can facilitate cell adhesion and proliferation, vascularization, and osteogenesis, thus accelerating and improving bone regeneration.^41,42^ In this study, we have turned our attention to the nanosilicate family, which is more stable than gelatin and easier to scale up compared to RGD modifications. Of the many inorganic nanosilicates investigated so far, Laponite (Na^+0.7^[(Mg_5.5_Li_0.3_) Si_8_O_20_(OH)_4_]^−0.7^) is the most appealing one due to its outstanding osteoconductivity.^43−46^ Laponite is a hydrous sodium lithium magnesium silicate that over time degrades into magnesium, orthosilicic acid, and lithium—minerals that subsequently can become incorporated by the cell nucleus to stimulate mesenchymal stem cells toward the osteogenic lineage.^45−50^ Notably, the positive charge on the rim surface of Laponite can form strong physical interactions with hydroxyl or carboxyl groups present in the alginate backbone,^50^ allowing users to incorporate it within the hydrogel matrix without tapping into complicated chemical conjugations.^51−53^ Nanosilicate implants have shown great potential as a biomaterial for orthopedic applications, thanks to their unique features, including excellent biocompatibility, the ability to degrade into harmless products, and osteoinductive properties. Their byproducts, such as orthosilicic acid, Mg^2+^, and Li^+^, which can be easily assimilated by the body, have been reported to promote osteogenic differentiation of human mesenchymal stem cells, making them a promising candidate for bone tissue engineering. The potential of nanosilicate implants as a growth-factor-free and cell-free microenvironment to accelerate bone healing is unique thanks to their significant osteoconductivity in vitro without differentiation factors, as demonstrated by expressing significant levels of osteogenic markers.^54^

One of the challenges associated with nanosilicates is assessing their toxicity, which is due to their degradation products. However, in physiological conditions, nanosilicates dissociate into nontoxic products such as Na^+^, Mg^2+^, Si(OH)_4_, and Li^+^. Nanosilicate implants have shown potential as a platform for drug delivery in tissue engineering due to their unique properties such as high surface area and biocompatibility. Nanoclays are commonly used in research related to tissue engineering, drug delivery, and wound healing because of their complete absence of toxicity. They can be easily engineered for drug loading and targeting and are highly biocompatible, nonimmunogenic, less expensive, and easily available. Nanoclays also possess unique properties such as intercalation, swelling, and nontoxic degradation products. The capacity for drugs to be loaded onto nanosilicate implants can vary due to a few factors, including the specific type of nanosilicate utilized, the shape and size of nanosilicates, and the specific drug that is being loaded onto it. Biomedically relevant and commonly used nanosilicates, such as kaolin, montmorillonite (MMT), and halloysite, are cationic clays with an overall permanent negative charge on the surface, allowing them to interact with basic drugs. For controlled drug release, drugs can also be intercalated between the nanosilicate layers. For instance, kaolin and halloysite have been successfully used to load doxorubicin (DOX) with a loading efficacy of approximately 54 and 80%, respectively. Laponite, due to its stacked structure, charges distribution over the surface, and no impurity has been commonly used as a drug delivery vehicle for DOX. MMT, one of the most studied natural nanosilicates for biomedical applications, is chemically resistant, stable under acidic conditions, and possesses an appreciable swelling capacity, making it a potent controlled release drug carrier.^55,56^

**Figure 1:**
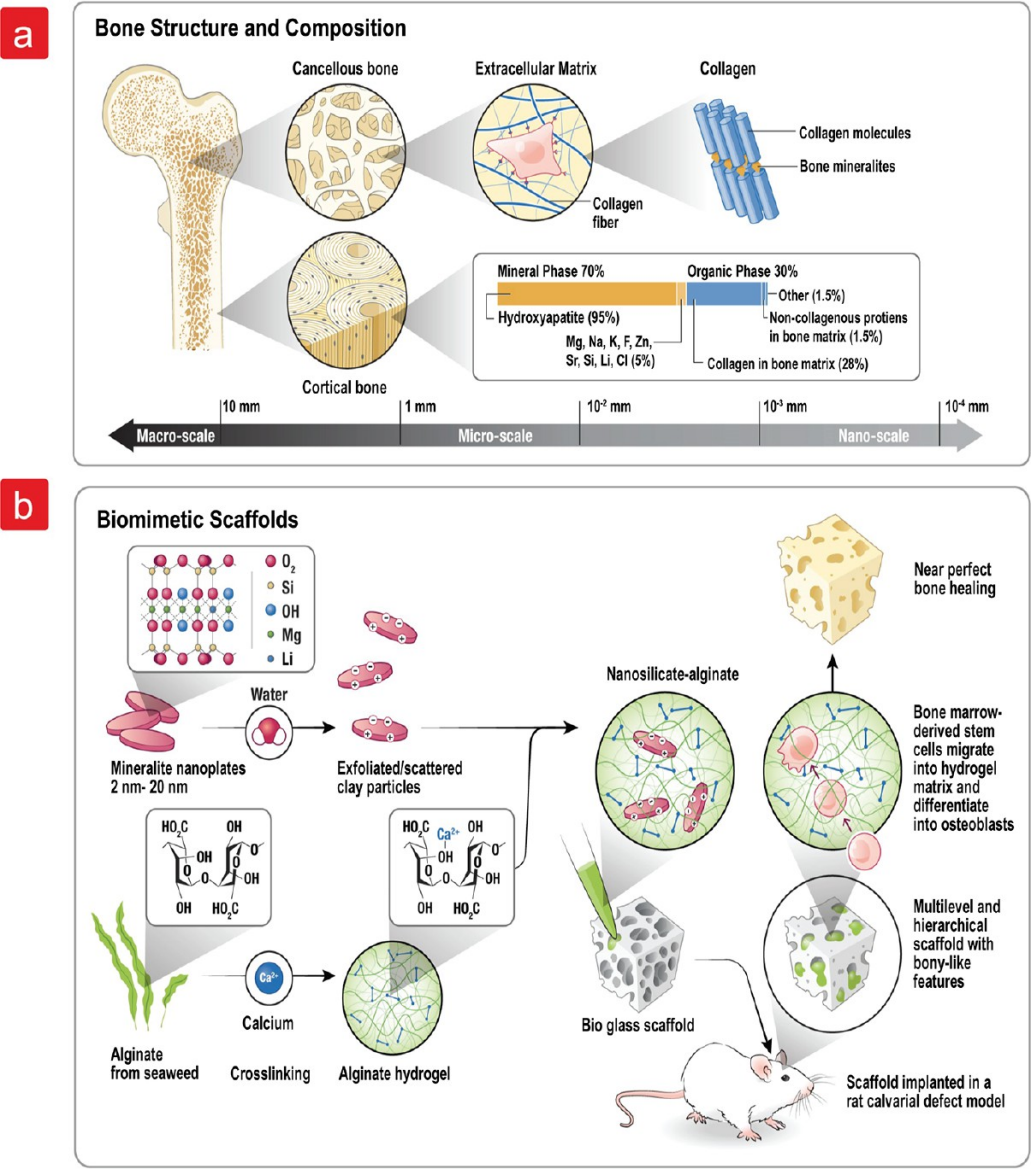
(a) Schematic representation of the (a) bone structure and its composition ranging from nanometers to micrometers and (b) development of biomimetic scaffolds containing the various steps for preparing a combinatorial hydrogel/scaffold.

Hydrogels and scaffolds have been used independently over the last two decades with their own lion’s share of advantages and shortcomings.^57−59^ Poor hydrogel mechanical strength makes them fail easily under the heavy loads usually present in the native bone, and the substantially harder porous scaffolds do not offer a native-like environment for cells to thrive within, resulting in insufficient bone formation. Combining the two into a single material could enable a trade-off to maintain their respective advantages while overcoming their shortcomings when used alone.^60,61^ For these reasons, scaffold–hydrogel systems should be at the very heart of the field in terms of recreating hard bone-like tissues.^62−70^ Unfortunately, only a few studies have examined the performance of these in vivo animal studies^63,67−70^ with poor outcomes accounting for a bone healing efficiency of about 5–45%^63,68−70^ after 1 month or 10.6–29.2%^69,70^ after 2 months. Indeed, even though mechanical requirements for optimal bone healing are fulfilled in the abovementioned studies, they did not mimic the complex hierarchical architecture of the native microenvironment, which contains features ranging from nanometer to micrometer, as well as the combinatorial chemistry of the mineral deposits within the native bone. For this reason, they resulted in suboptimal bone healing. Importantly, one of the long-standing challenges in bone tissue engineering, namely, the induction of progenitor cells toward the osteogenic lineage without differentiation growth factors, has not been addressed either in the abovementioned studies, and the recruitment and stimulation of native cells were minimal. However, what if one could address all of these issues by engineering a native-like multi-level scaffold consisting of a porous (>400 μm) hard inorganic phase embedding a soft ECM with nanoscale deposits in the form of mineral disks (2 nm)? In addition, what if the soft phase could induce a record-high bone formation by recruiting endogenous native cells and stimulate them toward the osteogenic lineage? The overarching goal of this study is exactly this, something we have accomplished by using a novel Laponite Alginate/58S BG-ceramic scaffold (60SiO_2_, 36CaO, 4P_2_O_5_ mol %) formulation that provides both a soft and hard phase with the capacity to recruit and stimulate native cells to form bone via the influence of Laponite (Figure 1).

Compared to unmodified scaffolds, this new scaffold led to improved osteoblast differentiation, expression of bone-related proteins, and upregulation of osteogenic genes, including collagen type I alpha 1 (COL1A1), osteopontin (OPN), and osteocalcin (OCN), even in the absence of differentiation factors. Notably, both histological staining and immunohistochemical assays showed the high capability of the cell-free scaffolds implanted in a rat calvarial bone defect to promote bone formation, with an almost complete bone healing reaching approximately 84%. Therefore, given its ability to modulate osteogenesis in a growth-factor-free environment and the high bone-forming ability intimately linked with it, we believe that we might finally have discovered a potential golden combination for repairing bone tissue.

## 2 Materials and Methods

### 2.1 Materials

Sodium alginate (SA, A2158, Sigma-Aldrich) and Laponite (BYK, USA) were utilized as starting materials for sample preparation. Calcium nitrate tetrahydrate (Ca(NO_3_)_2_·4H_2_O), triethyl phosphate, tetraethyl orthosilicate, nitric acid (HNO_3_), calcium chloride (CaCl_2_), polyvinyl alcohol (PVA), Triton X-100, Alizarin red S, dexamethasone, Dulbecco’s phosphate-buffered saline (DPBS), and Cell Counting Kit-8 (CCK-8) were purchased from Sigma-Aldrich. Diamidino-2-phenylindole (DAPI), phosphate-buffered saline (PBS) pH = 7.4, trypsin–EDTA, fetal bovine serum (FBS), 1% penicillin–streptomycin, horse serum and Eagle’s minimum essential medium (EMEM), and Dulbecco’s modified Eagle’s medium (DMEM) culture mediums were purchased from Thermo Fisher Scientific.

### 2.2 Cell-Laden Nanocomposite Hydrogel/BG Scaffold Preparation

#### 2.2.1 BG Scaffold Fabrication

58S BG was synthesized through the sol–gel method as described in our previous research.^15^ Briefly, a homogeneous slurry containing 40 wt % of BG powder, 30 vol % of ethanol, 30 vol % of deionized water, and 5 wt % of PVA was prepared after continuous mixing on a magnetic stirrer at 1000 rpm at 40 °C for 5 h. Furthermore, polyurethane foams (5 mm × 5 mm × 5 mm) were employed as sacrificial templates to make the scaffolds. The foams were dipped in a stable BG slurry, taken out, and dried overnight. To sinter the BG networks, the polymer foams were heat-treated at a heating rate of 2 °C/min at two different stages using an electrical furnace: (a) 450 °C/5 h (to burn out the sacrificial template)^71^ and (b) 800 °C/5 h (to consolidate and densify the BG structure).

#### 2.2.2 Cell-Laden Nanocomposite Hydrogel Preparation

Nanocomposite cell-laden hydrogels were prepared using alginate and Laponite through a simple design and mixing procedure in the presence of calcium chloride. Briefly, a stock solution of 3% (w/v) alginate was prepared by mixing alginate in deionized water using a magnetic stirrer. Laponite 1% (w/v) was mixed with deionized water, and the solution was magnetically stirred overnight to disperse and exfoliate Laponite sheets. Afterward, Laponite solution was slowly added to the alginate solution and allowed to mix properly under constant stirring at room temperature to reach a final concentration of 1.5% (w/v). Finally, rat bone marrow-derived MSCs (rBMSCs) were encapsulated within the 1% Laponite–alginate hydrogels (LH) at a density of 1 × 10^6^ cells mL^–1^ by mixing with the prepared solutions.

#### 2.2.3 Cell-Laden Nanocomposite Hydrogel and Reinforcing Scaffold Combination

The prepared cell-laden Laponite–alginate solution was gently injected into the hydrophilic porous BG to form combinatorial BG scaffold/cell-laden 1% Laponite–alginate hydrogel scaffolds (BGH). To crosslink the dispensed cell-laden hydrogels in the BG, an ionically crosslinking procedure was used. This was done by dipping the combinatorial cell-laden scaffolds for 15 min in 2% calcium chloride (CaCl_2_) solution. After crosslinking, all samples were rinsed with DPBS thrice and were then transferred into a 24-well plate filled with a complete medium. Finally, the samples were incubated at 37 °C in a humid 5% CO_2_ incubator for further studies. A schematic of the fabrication process is presented in Figure 1.

In total, for all experiments, three categories of samples were fabricated and coded as follows: BG, cell-laden LH, and BGH referred to the BG scaffold, cell-laden 1% Laponite–alginate hydrogel, and combinatorial BG scaffold/cell-laden 1% Laponite–alginate hydrogel, respectively.

### 2.3 In Vitro Cell Culture

rBMSCs were used at passages 3–4 and cultured in DMEM supplemented with 10% (v/v) FBS and 1% (v/v) penicillin–streptomycin in an incubator at 37 °C with 5% CO_2_. The cell-containing solution was mixed with a complete medium, transferred to a cell culture plate, and incubated at 37 °C in a humid 5% CO_2_ incubator. When the adherent cells became 80–90% confluent, they were sub-cultured with a warmed trypsin solution. In brief, they were washed two times with warmed PBS, and then trypsin solution was added, followed by incubation for 3 min. For further cell culture, the detached cells were cultured in growth media onto the culture plate at a proper density. Before cell seeding, the scaffolds were sterilized with 70% ethanol under ultraviolet light for 2 h and then washed three times with sterile PBS.

Overall, for each cellular test, the following four groups were considered: (1) BG, (2) cell-laden LH, (3) BGH, and (4) tissue culture polystyrene plate (TCP) considered as the control group. All the three scaffold groups were placed in a 24-well plate and incubated with the 500 μL complete medium. The BG group was placed in the first row, and the cells were seeded onto them at a density of 1 × 10^6^ cells mL^–1^; the cell-laden LH and BGH groups in which the cells were encapsulated in them were placed in the second and third rows, respectively, and the fourth row was assigned to the TCP group in which the cells were seeded in the culture well plate. The culture medium was changed with either complete (−) or differentiation culture media (+) every 2 days.

For osteogenic differentiation assays (i.e., alkaline phosphatase (ALP) activity and staining, Alizarin red S), cells at a higher density of 2 × 10^6^ cells mL^–1^ per hydrogel and scaffold were seeded onto scaffolds and encapsulated in hydrogels in a differentiation medium consisting of DMEM supplemented with FBS, dexamethasone, 1β-glycerophosphate, ascorbate-2-phosphate, and antibiotics. Scaffolds that were cultured in growth and differentiation media were considered as negative and positive samples, respectively.

### 2.4 Characterization

#### 2.4.1 Scanning Electron Microscopy and Energy-Dispersive X-ray Analysis

The morphology and microstructure of lyophilized scaffolds were observed using scanning electron microscopy (SEM: a FEI Quanta 200) equipped with an energy-dispersive X-ray spectroscopy (EDX) spectrometer. The scaffolds were rinsed in PBS thrice, frozen at −80 °C, and lyophilized for 48 h. The samples were then gold-sputtered (10 nm) and observed under SEM. The elemental distribution in the scaffolds was evaluated by the EDX spectrometer.

#### 2.4.2 Porosity Analysis

The scaffold porosity was calculated using the Archimedes principle. The scaffolds were taken in triplicate at a size of 10 × 10 × 10 mm^3^ and immersed in deionized water. Porosity (*P*) was defined as eq 1.
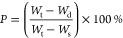
1where *W*_d_ is the dry weight of the scaffolds, *W*_s_ is the weight of the scaffolds suspended in water, and *W*_t_ is the weight of the scaffolds saturated with water.

#### 2.4.3 X-ray Powder Diffraction Analysis

To identify inorganic composition and determine the crystalline nature of BG and BG, their X-ray diffraction patterns were recorded after sample sintering using an X-ray diffractometer (Philips PW3040/60) with a Cu Kα radiation source (λ = 1.5405 Å) in the range of 2θ = 10–80° at a step size of 0.02°. The crystallinity of the samples was determined by dividing the integrated areas of crystalline peaks by the total integrated areas under the X-ray diffraction (XRD) peaks. In addition, the phase composition of in vitro tested scaffolds, as well as scaffolds before and after immersion in SBF, was evaluated by XRD analysis using the acquisition conditions stated before.

#### 2.4.4 Fourier Transform Infrared Spectroscopy Analysis

Fourier transform infrared spectroscopy (FTIR, PerkinElmer Frontier, USA) was used to confirm the chemical functional groups of Laponite and alginate in BGH. The transmittance spectra of the lyophilized hydrogel, the BG scaffold, and the combinatorial scaffold loaded with the Laponite–alginate hydrogel were recorded at 4000–500 cm^–1^.

#### 2.4.5 Ion Release Evaluation

To study the release profile of ions including Ca, P, Si, Mg, Li, and Na from both BG and BGH up to 28 days of soaking in SBF solution, inductively coupled plasma optical emission spectroscopy (ICP-OES, Varian, Vista-MPX) was used. The samples were kept in polyethylene bottles containing SBF in an incubator at 37 °C under static conditions, and the ion release was evaluated at 1, 3, 5, 7, 14, 21, and 28 days. The mass-to-volume ratio was 1.5 mg mL^–1^. The concentration of ions was determined by analyzing aliquots of the various solutions collected at each time point.

#### 2.4.6 Rheological Measurements

The rheological properties including storage modulus (*G*′) and viscosity of the 1% Laponite–alginate solution (LA) were evaluated before and after the crosslinking with 2% CaCl_2_ for 3 min by a rheometer (TA Instrument, USA) supplemented with plate geometry (25 mm in diameter) with a gap distance of 200 μm. The storage modulus (*G*′) was measured using time sweep test, and the shear-thinning properties were investigated using flow sweep tests by monitoring viscosity curve versus shear rate under frequency conditions of 1 Hz and a shear rate of 0.1–102 s^–1^ at 25 °C.

#### 2.4.7 Mechanical Characterization

The compressive properties of the scaffolds were investigated using a (Santam, stm20, Korea) universal testing machine equipped with a 100 N load cell at a rate of 1 mm s^–1^. Scaffolds with dimensions of 10 × 10 × 10 mm^3^ were tested at room temperature both in air (dry) and in wet conditions. Thus, prior to the compression test, the scaffolds were rehydrated with PBS overnight at 37 °C for wet conditions. The compressive strength and Young’s modulus of scaffolds were determined by the compression test using at least three replicates. The compressive strength of the specimens was calculated by dividing the maximum applied force by the cross-sectional area, and Young’s modulus was determined as the slope of a stress–strain curve at (0–0.1 of total strain).

#### 2.4.8 In Vitro Degradation Evaluation

To study the degradation behavior, the three groups of developed scaffolds including BG, LH, and BGH (*n* = 3 samples per group) were immersed in PBS (pH 7.4) and incubated at 37 °C for 30 days. The initial weight (*W*_i_) of the scaffolds was recorded. After the incubation periods, the scaffolds were removed from PBS, rinsed with deionized water, and air-dried at room temperature. The final dry weight of the scaffolds (*W*_f_) was measured at each time. Finally, the weight loss (%) of each group was quantified as eq 2.
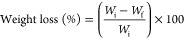
2

### 2.5 Cytocompatibility Studies

#### 2.5.1 Live/Dead Staining Assay

The cell viability was quantitatively evaluated using the live/dead assay (Thermo Fisher, USA) after 1, 7, and 14 days of cell culture. Following rinsing three times in PBS, the scaffolds were incubated for 45 min in DMEM supplemented with 2 mM calcein AM as well as 4 mM ethidium homodimer-1. A confocal laser scanning microscope (TE2000-S, Nikon, Tokyo, Japan) was used to record images of the stained cells after washing the scaffolds with PBS three times. Green cells were considered alive, while red cells were considered dead. Finally, the percentage of viable cells relative to the total number of cells found in each image was calculated to determine the cell viability (%).

#### 2.5.2 Cell Morphology

Morphology and adhesion of cells within the three groups of scaffolds were observed by SEM. Incubation of the scaffolds for 7 days was followed by cell fixation with glutaraldehyde solution of 2.5% (Sigma-Aldrich), followed by 10 min of ethanol dehydration (30, 50, 75, 90, and 100%). A 24 h freeze-drying process was performed on the scaffolds. Finally, a scanning electron microscope FEI Quanta 200 was used to observe the gold-coated scaffolds.

The nuclei of the incubated cells were visualized by using diamidino-2-phenylindole (DAPI) fluorescent staining. Briefly, the scaffolds were washed thrice with PBS after 14 days of cell culture and then fixed in a formaldehyde solution of 4% (Thermo Fisher, USA) for 15 min at room temperature. In order to permit membrane permeabilization, the scaffolds were then incubated in PBS containing 0.1% (v/v) Triton X-100 (Sigma-Aldrich) for 10 min. Afterward, the scaffolds were rinsed two times with PBS and incubated in DAPI solution in a dark state at 37 °C for 30 min, followed by three gentle washes with DPBS, and images were taken with a confocal laser scanning microscope (Nikon TMS). Nuclei counting within the scaffolds were evaluated for at least four images per scaffold and analyzed using ImageJ software.

#### 2.5.3 Immunocytochemical Staining

After 21 days of in vitro culture, the cells were washed three times with PBS and fixed with 4% paraformaldehyde for 20 min at 4 °C. The cells were incubated in 0.3% TritonX-100 in PBS for 30 min and then blocked with 1% BSA in PBS for 30 min at room temperature. Afterward, osteopontin (OPN) and osteocalcin (OCN) staining were performed by incubating the scaffolds containing fixed cells with primary antibodies, anti-OPN (ab8448, 1:100; Abcam), and anti-OCN (ab13418, 1:100; Abcam) diluted in PBS containing overnight at 4 °C. After three times washing with PBS, the scaffolds were incubated in the secondary antibody (Alexa flour 488-conjugated, 1:150; Abcam) at room temperature for 1 h in a dark place. The samples were again washed with PBS three times, and finally, the nuclei were stained with a 1:500 diluted DAPI solution. The samples were washed with PBS three times, and the images were captured and analyzed under a fluorescent microscope (Olympus Corporation, Tokyo, Japan).

#### 2.5.4 Real-Time-PCR Analysis

To quantitatively analyze the osteogenic differentiation of rBMSCs on BG, cell-laden LH, and BGH scaffolds, real-time (RT)-PCR was performed on day 14. To this end, the gene expression of bone osteogenic markers, including collagen type I alpha 1 (COL1A1), osteopontin (OPN), and osteocalcin (OCN), was assessed by the RT-PCR technique assay. Briefly, total RNA was isolated from the cells cultured on scaffolds using the TRIzol reagent (Invitrogen, USA) and then synthesized to cDNA according to the instructions using the PrimeScript RT Master Mix. RT-PCR was performed to determine the gene-level expression of COL1A1, OPN, OCN, and glyceraldehyde-3-phosphate dehydrogenase (GAPDH) as the housekeeping gene (Table 1) using the SYBR Green PCR master mix (Applied Biosystems Life Technologies). All the samples were tested in triplicate, and the expression levels of all genes were normalized relative to GAPDH and measured using the comparative 2^–ΔΔ*Ct*^ method. The sequences of primers used in this study are listed in Table 1.

**Table 1:** qRT-PCR Primer Sequences for Rats

gene	forward primer	reverse primer	product size (bps)	accession number
COL1A1	GAATATGTATCACCAGACGCAG	AGCAAAGTTTCCTCCAAGAC	186	NM_053304.1
OPN	GAGGAGAAGGCGCATTACAG	GTCATCGTCGTCGTCATCAT	198	XM_008769996.2
OCN	GAGGGCAGTAAGGTGGTGAA	GTCCGCTAGCTCGTCACAAT	135	NM_013414.1
GAPDH	GAAACCTGCCAAGTATGATGAC	CATTGTCATACCAGGAAATGAGC	200	NM_017008.4

#### 2.5.5 ALP Staining and Activity

ALP staining was performed to determine the intracellular ALP activity of the rBMSCs using a BCIP/NBT (5-bromo-4-chloro-3-indolyl phosphate/nitro blue tetrazolium, Thermo Scientific, USA) solution. Briefly, after 14 days of cell culture, the scaffolds were removed and washed twice with PBS, stained with BCIP/NBT, and incubated in the dark at room temperature for 2 h. The reaction was then stopped by discarding the excess BCIP/NBT staining solution and gently rinsing three times with PBS. Images were taken under an optical microscope. The ALP expression was determined from the area surrounded by the purple-stained cells.

Moreover, to confirm the osteogenic differentiation that was indicated by ALP staining, the ALP activity of the rBMSCs was assayed using the ALP assay kit (ab83369, Abcam, United Kingdom). After 7 and 14 days of osteogenic induction of the MSCs on the scaffolds, they were rinsed gently thrice with PBS. The scaffolds were first treated with a 1.6 M sodium citrate solution for 2 h at 37 °C to degrade the hydrogels by breaking the ionic crosslinks and then permeabilized in a 10 mM Tris buffer containing a 0.1% Triton X-100 solution at room temperature. Afterward, the solution was centrifuged for 2 min at 4 °C. Subsequently, 50 μL of the supernatant was mixed with 100 μL of lysates and added to each well containing 50 μL of pNPP solution prepared using an ALP kit (Sigma-Aldrich) and incubated at 37 °C for 2 h. The conversion of *p*-nitrophenyl phosphate into *p*-nitrophenol was assessed by measuring the absorbance (OD) of the reacted sample solution at 405 nm using a microplate reader Tecan Infinite M2000.

#### 2.5.6 Alizarin Red S Staining and Quantitative Assay

Calcium deposition was examined by staining rBMSCs cultured on three groups of scaffolds after 2 and 3 weeks of culture with Alizarin Red S (ARS). In brief, the scaffolds were fixed in a 4% paraformaldehyde for 15 min. Then, the scaffolds were rinsed in PBS twice, followed by adding 1% Alizarin red S solution for 30 min in a light-protected environment at room temperature. After staining, the scaffolds were washed repeatedly with PBS to remove any further color, and the mineralized nodules after 3 weeks of culture were then imaged using an optical microscope. Additionally, the calcium deposition was quantified by ARS staining. 10% Cetylpyridinium chloride (Sigma-Aldrich Co., USA) was added to the scaffolds for 15 min at room temperature to extract the staining. Finally, the absorbance (OD) was measured at 562 nm using a microplate reader (Tecan Infinite M2000). All data values are defined as means ± standard deviation (SD) (*n* = 5).

#### 2.5.7 In Vitro Mineralization

The in vitro bioactivity of the scaffolds was examined by the rate of apatite-forming ability during their immersion in simulated body fluid (SBF). The SBF solution was prepared according to Kokubo’s procedure.^21^ The three groups of scaffolds including BG, LH, and BGH were immersed in SBF for up to 14 days, then removed from the SBF solution and washed with deionized water, and lyophilized for 48 h. Finally, the apatite formation on the surface of scaffolds was characterized using SEM, XRD, and FTIR.

#### 2.5.8 In Vivo Bone Regeneration

Animal experiments were carried out on a rat calvarial bone defect model to evaluate the potential capability of various scaffolds to promote in vivo bone regeneration. All animal experiments were approved and performed according to the regulations for animal experiments of the University Animal Ethics Committee of Tarbiat Modares University. The surgeries were performed in 12 healthy, 8 week male Wistar rats weighing 250 g, obtained from Pasteur Institute of Iran. The animals were randomly divided into four groups: (1) control group (empty defects), (2) cell-free BG, (3) LH, and (4) BGH scaffolds (three animals/group). Surgical procedure was performed by first anesthetizing the animals using an intramuscular injection of two parts [ketamine (100 mg/mL) with xylazine (1%)], followed by shaving and disinfecting of the implantation regions. Then, an incision in the skin was made, and a circular defect with 5 mm in diameter was created on the left cranium of each rat using a trephine bur where scaffolds were implanted. All groups except the control group were implanted into the calvarial defect and, finally, the wounds were stitched with sutures. The animals were sacrificed after 8 weeks of implantation using CO_2_ asphyxiation, and the calvaria were retrieved for further analysis.

#### 2.5.9 Histological and Immunohistochemistry Staining

The harvested calvaria samples were fixed with 4% paraformaldehyde for 48 h at 4 °C and then dehydrated with a graded ethanol series. Afterward, the samples were decalcified using 0.5 M ethylenediaminetetraacetic acid (EDTA) for 2 weeks and embedded in paraffin blocks. Paraffin sections with 5 μm thickness were prepared using a microtome to demonstrate the defect with the surrounding bone regeneration. The slides were then stained with hematoxylin–eosin (H&E) and Masson’s trichrome and visualized under an optical microscope. To quantify the newly formed bone, the histological images were assessed via ImageJ based on the difference in the threshold, and the mean newly formed bone area to total area in each group was determined. For the immunohistochemistry staining, the samples were washed with PBS, followed by permeabilization with 0.3% Triton X-100 for 30 min, and then blocked with 10% BSA at room temperature for 30 min before incubated in primary antibodies including osteopontin (OPN, ab8448, 1:100; Abcam) and osteocalcin (OCN, ab13418, 1:100; Abcam) at 4 °C overnight. After being washed with PBS, the secondary antibody (Alexa flour 488-conjugated, 1:150; Abcam) was applied for 1:30 h at room temperature. Afterward, the samples were washed with PBS three times in a dark place, and the nuclei were stained with DAPI, followed by washing with PBS. Finally, the samples were observed under a fluorescent microscope (Olympus Corporation, Tokyo, Japan) and analyzed using ImageJ software to quantify the positive reaction area.

### 2.6 Statistical Analysis

Statistical analysis was carried out using GraphPad Prism 6 software. Error bars are plotted as mean ± SD. All samples were tested in triplicate unless otherwise noted. Statistical comparison was examined by one-way analysis of variance (ANOVA), followed by a post hoc Tukey’s test. Finally, the statistical significance was determined as *p < 0.05, **p < 0.01, ***p < 0.001, and ****p < 0.0001.

## 3 Results

### 3.1 Microstructural Characterization

**Figure 2:**
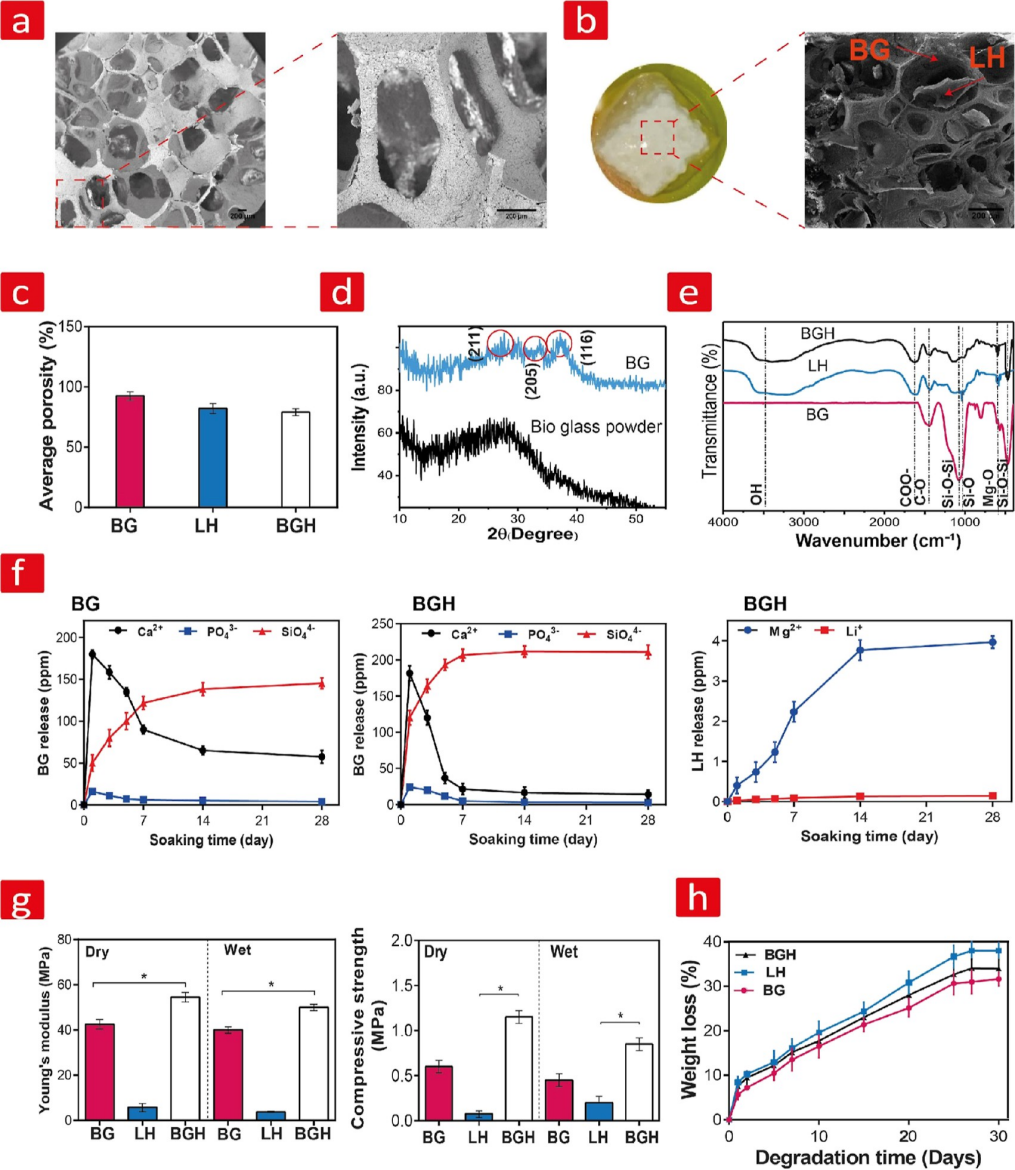
Microstructural, chemical, mechanical, and degradation analysis. (a) SEM images of pores and strut structures of BG. (b) Optical and SEM images of the developed BGH scaffold. (c) Average porosity of BG, LH, and BGH scaffolds. (d) XRD patterns of BG powder and BG. (e) FTIR spectra of the developed BG, LH, and BGH. (f) Concentration of released minerals including Ca^2+^, SiO_4_^4–^, and PO_4_^3–^ from BG and BGH, as well as Mg^2+^ and Li^+^ release from LH. (g) Compressive strength and compressive modulus of BG, LH, and BGH in wet and dry conditions. (h) In vitro degradation of the three groups after 30 days in PBS. Statistical significance: *p < 0.05.

A pore size of around 300 μm in combination with high interconnectivity and porosity is important to consider in scaffold design since they can enable efficient nutrient and waste exchange as well as most importantly facilitate vascularization. Without this, a necrotic core will develop over time, leading to implant failure.^72,73^ For this reason, the surface and cross-section of our scaffolds have been characterized with SEM and depicted in Figure 2a,b. The SEM images show porous structures with interconnected open pores, homogeneous pore sizes, and struts. The average pore size of the BG scaffold was found to be around 451.7 ± 40 μm, while that of 1% Laponite–alginate hydrogel (LH) and combinatorial BG scaffold/1% Laponite–alginate hydrogel (BGH) was 110.6 ± 35 and 414.3 ± 20 μm, respectively. From the images, we can also see that LH was successfully loaded on the harder BG scaffold. Additionally, the SEM image indicated that the observed structural integrity, interconnectivity, and porosity of BG also prevailed here. Furthermore, the porosities of BG and BGH measured by the Archimedes method are shown in Figure 2c. Here, BG displayed a porosity of about 92.5 ± 3.5%, while the porosity of LH decreased to 82 ± 3.7% and that of BGH to 79 ± 2.8%. The drop observed for BGH is most likely related to the lesser porous LH composite filling it out. In summary, we can conclude that the scaffolding systems employed herein are sufficiently porous and interconnected and thus not a compromising factor in the tissue regeneration process.

### 3.2 Chemical Analysis

The crystallographic nature of BG was determined using XRD and is displayed in Figure 2d. The BG powder showed an amorphous structure characterized by a wide band between 15 and 30°.^15^ The XRD pattern of the heat-treated BG scaffold on the other hand showed a semicrystalline structure with a crystallinity value of 52.3% as calculated from the XRD data and with diffraction peaks at 2θ values of 30, 32, and 37°. These peaks match with the known diffraction pattern of dicalcium silicate (Ca_2_SiO_4_).^74^ Therefore, the crystalline nature of BG is primarily made from Ca_2_SiO_4_. From FTIR spectrum analysis, we observed that BG exhibited characteristic peaks of Si–O–Si bending and stretching vibrations at 467 cm^–1^ and 819 and 1067 cm^–1^, respectively (Figure 2d). Moreover, the presence of P–O bending vibrations was accounted for by a peak at 570–620 cm^–1^.^75^ Overall, this together with the XRD data confirms that the chemical structure of BG is dominated by calcium, silicate, and phosphate—mineralites that are essential for securing high bone well-being. On the other hand, the presence of Laponite within LH was confirmed by peaks at 640 and 984 cm^–1^, which are associated with Mg–O vibrations and the Si–O stretching bands, respectively. The peaks related to alginate were also observed at 1411 and 1594 cm^–1^, which correspond to the symmetric and asymmetric stretching vibrations of carboxylate bonds (COO−), respectively. A broad O–H stretching vibration peak corresponding to the hydroxyl group of alginates was also observed at 3200–3600 cm^–1^.^76−79^ Last, FTIR analysis of BGH showed that peaks related to the carboxylate bonds (COO−) of alginate shifted to higher wavelengths at 1416 and 1601 cm^–1^ (Figure 2e). This might be due to the interactions of carboxylic groups in alginate with the silica groups of Laponite leading to the formation of hydrogen bonds (Si–O–Si) in BGH. Moreover, the combination of BG and LH was confirmed by the presence of overlapping peaks from BG (P–O) and LH (Mg–O). Two strong characteristic peaks of Laponite were shifted as well toward higher values of 645 and 999 cm^–1^, indicating the existence of physical hydrogen bonds such as OH binding to MgO and Si–O.

### 3.3 Minerals Release Study

The concentrations of Ca^2+^, SiO_4_^4–^, and PO_4_^3–^ released from BG and the concentration of released Laponite mineral constituent ions including Mg^2+^ and Li^+^ from BGH were determined through an ICP assay (Figure 2f). The release of Na^+^ is presented in Figure S1 (Supporting Information). The results demonstrated that the concentration of SiO_4_^4–^ increased over time in contrast to the concentration of Ca^2+^ and PO_4_^3–^, which increased initially, after which they began to decrease due to the consumption of Ca^2+^ and PO_4_^3–^ ions during the process of hydroxyapatite [HA, hydroxyapatite Ca_10_(PO_4_)_6_(OH)_2_] formation on the scaffolds. The decrease of Ca^2+^ and PO_4_^3–^ for BG reached a constant value after 7 days, while this reduction for BGH reached a constant value after 5 days due to the faster release of ions and, therefore, a higher HA formation rate. This could be related to the observed higher degradation rate of BGH, as observed in Figure 2h. Moreover, the results indicated that after 28 days, the concentration of Ca^2+^ and PO_4_^3–^ ions released from BGH was lower than that for the BG. This can be attributed to the presence of Laponite since Laponite itself has the ability to form HA on its surface due to the fact that SiO_4_^4–^, Mg^2+^, Li^+^, and Na^+^ ions can be released from Laponite and accelerate this process.^80^ In summary, BGH is truly combinatorial as it can release a complex mixture of ions with concentrations ranging from 145.33 ppm (SiO_4_^4–^), 57.45 ppm (Ca^2+^), 4 ppm (PO_4_^3–^) from the hard BG phase and 4 ppm (Mg^2+^) and 0.14 ppm (Li^+^) from the softer LH phase. This is indeed a noteworthy finding since numerous studies have hypothesized that they both on their own and in combination with other mineralites can play a significant role in osteoblast activities and facilitate osteogenesis without the aid of differentiation factors.^18,45,46,51^

### 3.4 Rheological, Mechanical, and Degradation Studies

#### 3.4.1 Rheological Properties

The rheological properties of LA solution before and after ionic crosslinking with CaCl_2_ were assessed by measuring its viscosity and storage modulus (Figure S2, Supporting Information). Figure S2a shows the flow curve over a range of shear rates (0.1–100 s^–1^) for both LA and LH without and with the addition of CaCl_2_. As shown in Figure S2a, the viscosity decreased with shear rate, and a shear-thinning behavior was observed for both samples. It was concluded that the ionic crosslinker increased the viscosity of LH from 22 to 390 Pa s (Figure S2a), and the *G*′ of LH solution increased similarly from 26 to 1065 Pa (Figure S2b, Supporting Information) after approximately 3 min, which suggests that the crosslinking of LH is sufficiently long for proper handling during the cell encapsulation stage.

#### 3.4.2 Mechanical Properties and Degradation Study

The mechanical properties of implants are at the center of their function within native bone and, therefore, considered an important parameter in bone tissue engineering.^12^ Scaffolds intended for use within defects in cancellous bone need to exhibit sufficient compressive strength and modulus usually between 0.1–16 MPa and 0.05–0.5 GPa, respectively, to match that of the native milieu.^81,82^ For this reason, we have examined the mechanical properties of BG, LH, and BGH. We specifically focused on compressive strength and compressive modulus in both dry and wet conditions (Figure 2g). The compressive strength and compressive modulus of BGH were about 1.15 ± 0.07 and 39 ± 1.41 MPa in dry and 0.85 ± 0.07 and 33.5 ± 2.12 MPa in wet conditions, respectively, both matching with the mechanical properties of human cancellous bone.^81,82^ The compressive strength of BG decreased from 0.6 ± 0.071 MPa in dry conditions to 0.45 ± 0.07 MPa in wet conditions, while the compressive modulus decreased from 32 ± 1.41 to 28.5 ± 2.12 MPa. On the other hand, the compressive strength of LH on the other hand decreased from 0.2 ± 0.07 to 0.075 ± 0.035 MPa in wet conditions. The same trend was also reported for the compressive modulus, which decreased from 5.75 ± 2.47 to 3.75 ± 1.76 MPa in wet conditions. A weaker interaction most likely causes this among inorganic and organic ingredients due to substantial hydrogel swelling in a wet condition caused by ion exchange in which ions from media replace the Ca^2+^ alginate crosslinker.^83^ An important reason for the higher mechanical values found for the combinatorial BGH scaffold can be attributed to its pores and porosities which we examined earlier via SEM imaging and the Archimedes method because the mechanical properties of the scaffolds strengthen by a drop in pore size and the pore size of BGH dropped to 414.3 ± 20 μm compared to BG and LH (451.7 ± 40 μm and 110.6 ± 35, respectively), which in turn could explain the higher compressive properties. Moreover, the inclusion of LH into BG also increases the material density and thus the compressive properties of BGH (BG + LH).

We then studied the in vitro degradability behavior of the three groups to assess whether they have sufficient stability for downstream wet experiments (Figure 2h). The results indicated that after 30 days in PBS, the degradation of LH was highest with a reported weight loss at 38 ± 3%, while the degradation of BGH was slightly lower (34 ± 2%) but still higher compared to that of BG (31.66 ± 1.52%) (Figure 2h). The presence of LH within BGH could explain the higher degradation observed here compared to BG. Overall, the results demonstrated that BGH displayed sufficient stability for wet experiments, thereby providing a sustainable environment for cell encapsulation studies.

### 3.5 Cell Adhesion and Viability

#### 3.5.1 Cell Adhesion Behavior

The attachment and morphology of rat bone marrow-derived MSCs (rBMSCs) on BG, encapsulated within LH, and combinatorial BGH scaffolds were investigated with SEM as shown in Figure S3a (Supporting Information). The red arrows show well-adhered rBMSCs—something observed on all groups—whereas the black arrows point to the direction of the Laponite–alginate hydrogel. In more detail, one could see that cells on BG exhibited a flattened morphology. On the other hand, rBMSCs cultured within LH showed a more rounded morphological shape. This could be related to the fact that they were encapsulated within LH and, therefore, more restricted mobility-wise. Indeed, as shown in Figure S3a, LH rBMSCs were surrounded by the alginate hydrogel (whitish and translucent in the images). On the other hand, we can see from the SEM images that the rBMSCs in the combinatorial BGH scaffold were extended and extremely stretched on the boundary between struts and pores. After 14 days of incubation, the rBMSC number distribution in each group was determined by counting the cell nucleus using DAPI staining (Figure S3b). The highest cell density was observed on BGH (75 cells mm^–3^), while cell-laden LH and BG gave 63.33 and 56.33 cells mm^–3^, respectively (Figure S3c, Supporting Information). We found a fairly even cell count in all groups. However, there were fewer cells that resided on BG compared to cell-laden LH and BGH. Overall, we can conclude a better adhesion of rBMSCs on the combinatorial BGH scaffold compared to BG and cell-laden LH samples. The reason can be attributed to the more native-like environment present on BGH due to the ECM-like resemblances of the nanosilicate hydrogels both in terms of softness and water content.

#### 3.5.2 Cell Viability

The effectiveness of biomaterials largely depends on how they biologically react with human tissues, which is known as biocompatibility. Bioceramics are an appealing option for biological implants because they exhibit high biocompatibility, resulting in minimal tissue reaction, low toxicity, and no risk of inflammatory or allergic reactions. They are chemically stable within the biological environment and do not shrink. Two key factors that affect biocompatibility are the response of the host tissue to the material and the material’s degradation in the body, which bioceramics do so safely, degrading into nontoxic inorganic products. Bioceramics are commonly utilized in biomedical applications, including orthopedic and dental implants, as well as drug delivery systems, due to their excellent biocompatibility and ability to promote cell viability and tissue repair.^84−86^ Viability is considered an important factor in scaffold biocompatibility evaluation because it can affect cell growth and differentiation. Therefore, cell viability and the number of cells were qualitatively evaluated by live/dead staining. Figure S4a shows confocal images of all the three groups after 1, 7, and 14 days, demonstrating a higher number of live (green) cells in all groups on day 14 than on days 1 and 7 (Figure S4a, Supporting Information). The number of live cells in BGH was greater than BG at all time points. Importantly, compared to BG, almost no dead cells were observed here after 14 days. The cell viability was subsequently calculated from these images for each group (Figure S4c, Supporting Information) by dividing the number of live cells by the total number of cells (live and dead). The calculations showed that all samples could maintain high cell viability (>85%). Indeed, after 14 days of cell culture, the cell viability was 85.9 ± 3.74, 89.4 ± 7.02, and 100 ± 3.53% for BG, cell-laden LH, and BGH scaffolds, respectively. We can thus conclude that BGH indicates excellent biocompatibility and supports higher cell viability than the other combinations, which could be attributed to the extremely hydrated environment provided by the embedded nanosilicate hydrogel, enabling it to quickly absorb and keep cell media over longer time points.

### 3.6 Osteogenic Differentiation and Biomineralization Study

#### 3.6.1 In Vitro Osteogenic Activity

**Figure 3:**
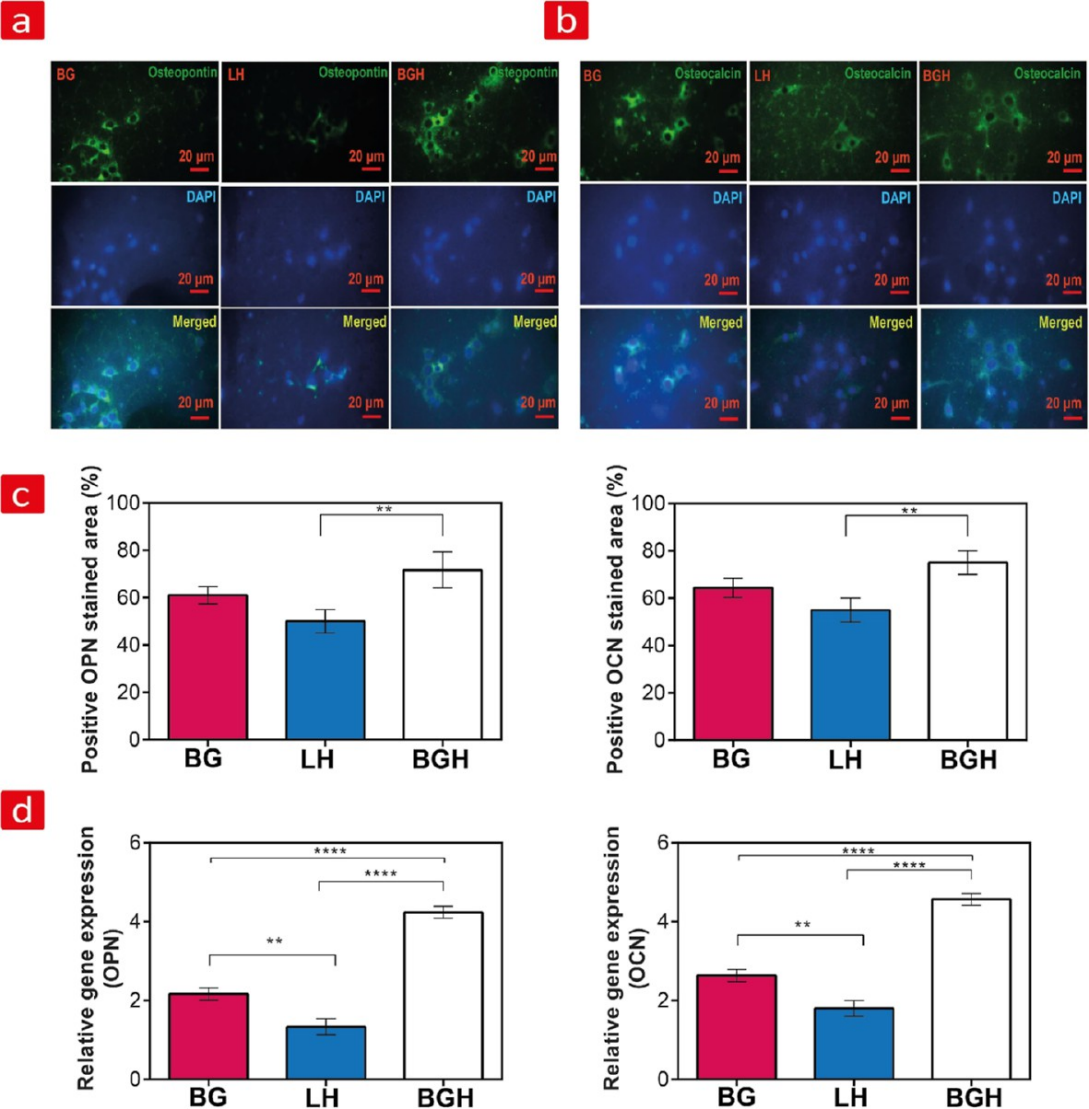
Osteogenic gene expression. (a) OPN and (b) OCN protein expression from rBMSCs seeded on BG, cell-laden LH, and BGH after 21 days of in vitro incubation. OPN and OCN are visible by green fluorescence and the nuclei with blue. (c) The OPN- and OCN-stained areas calculated from the fluorescence images are shown here. (d) OPN and OCN gene expression from rBMSCs seeded on BG, cell-laden LH, and BGH after 14 days of incubation, **p < 0.05: statistically significant differences. (One-way ANOVA was used; **p < 0.05 and ****p < 0.0001.)

The expression of mid-stage bone differentiation markers, including OPN and OCN, after 21 days of rBMSC culture on BG, cell-laden LH, and BGH was assessed by immunocytochemistry staining (Figure 3a,b). The positive green stained area confirmed the expression of both proteins in all the three groups, demonstrating that rBMSCs underwent osteogenic differentiation. Notably, the expression of OPN and OCN on BGH seemed more pronounced than BG and cell-laden LH groups (Figure 3a,b). Additionally, quantitative analysis of fluorescence-positive areas indicated a similar trend (Figure 3c). From here, we estimated the OPN- and OCN-stained area values of BGH as 71.66 ± 7.63 and 75 ± 5, respectively, which was significantly higher than that of BG (61 ± 3.6 and 64.33 ± 4, respectively) and cell-laden LH (50 ± 5 and 55 ± 5, respectively) groups. Overall, the results confirmed that the combinatorial BGH scaffold exhibits higher osteogenesis in vitro than BG and LH.

#### 3.6.2 Real-Time PCR

To follow up on the immunocytochemistry results in the previous section, we examined the expression levels of OPN and OCN on a genetic level via RT-PCR at day 14 (Figure 3d). We also examined the COL1A1 gene expression after 14 days of osteogenic induction, as evident from the supplementary section (Figure S5, Supporting Information). OPN, OCN, and COL1A1 were upregulated on all formulations after 14 days of incubation. However, BGH showed a higher expression level of COL1A1 (3.63 ± 0.15), OPN (4.23 ± 0.15), and OCN (4.6 ± 0.25) compared to the BG [COL1A1 (1.83 ± 0.15), OPN (2.16 ± 0.15), and OCN (2.63 ± 0.15)] and cell-laden LH [COL1A1 (1.26 ± 0.15), OPN (1.33 ± 0.2), and OCN (1.8 ± 0.2)] groups (Figure 3d). In other words, rBMSCs cultured on the BGHs expressed a higher level of COL1A1, OPN, and OCN than those in other groups (****p < 0.0001). Between BG and cell-laden LH, a higher expression of bone-related genes can be seen in the BG (**P < 0.05).

#### 3.6.3 ALP Expression and Activity

**Figure 4:**
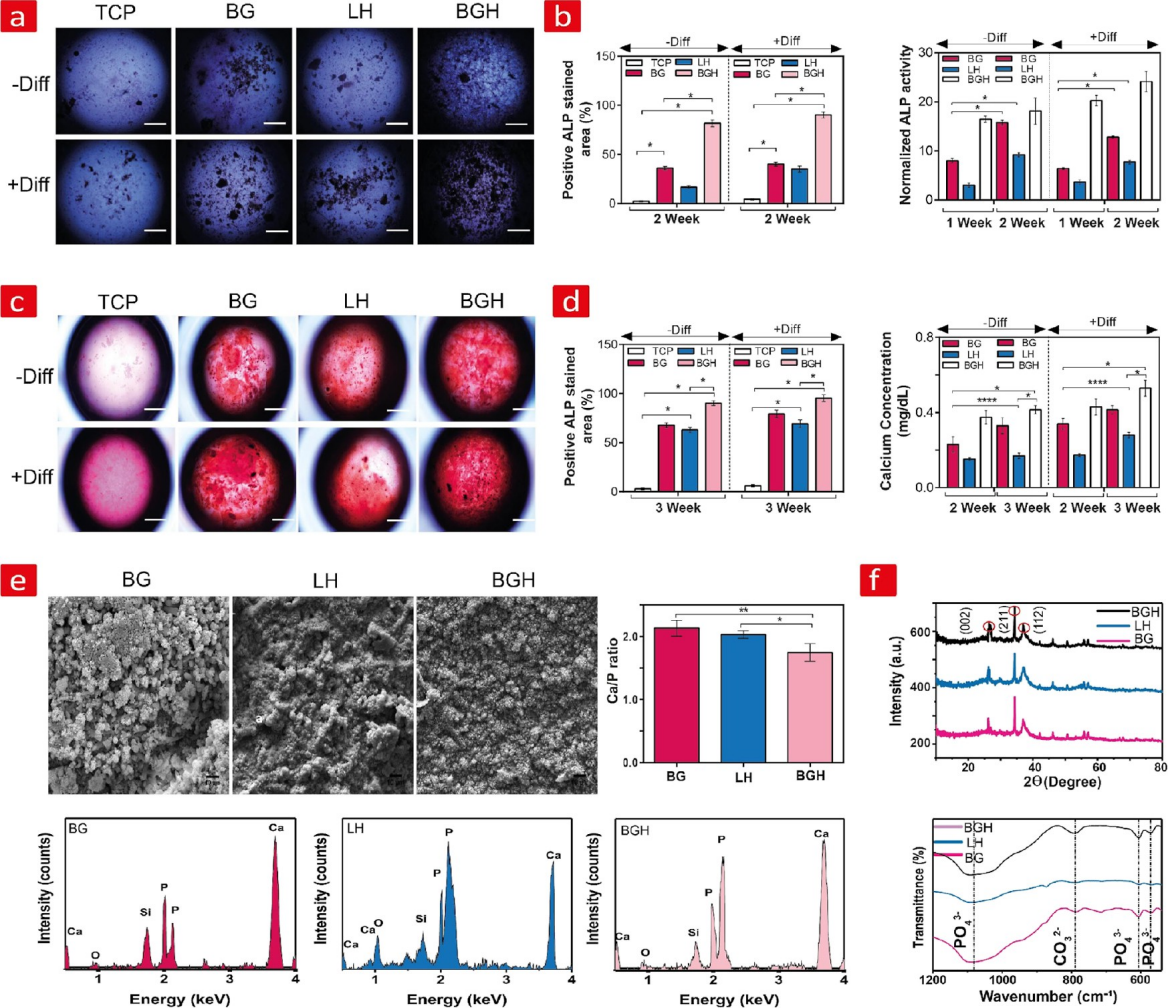
In vitro cell differentiation and biomineralization studies. (a) Bright-field images of ALP-stained samples after 2 weeks. (b) The positive ALP-stained area and the ALP activity within the scaffolds were measured after 1 and 2 weeks. (c) Bright-field images of the Alizarin Red S stained samples after 3 weeks. (d) The positive Alizarin Red S-stained area and the calcium concentration were measured on TCP, BG, cell-laden LH, and BGH scaffolds after 2 and 3 weeks in the absence and presence of differentiation media via Alizarin red assay and calcium assay. (e) SEM images, Ca/P ratio, and EDX analysis of the BG and BGH scaffolds after immersion in SBF for 14 days. (f) XRD patterns and FTIR spectra of the scaffolds after immersion in SBF for 14 days. Peaks related to the formation of hydroxyapatite are marked with stars. *p < 0.05 and ****p < 0.001: statistically significant differences. Error bars represent the means ± SD.

ALP is an important early osteogenic differentiation marker.^87^ We have, therefore, qualitatively and quantitatively investigated this marker after 1 and 2 weeks in the presence (+) and absence (−) of differentiation factors for all combinations via ALP staining (Figure 4a,b). In the presence of differentiation media (+), all groups expressed more ALP compared to differentiation media (−) as evident from the increased purple color density in the brightfield images (Figure 4a). Notably, all the three groups exhibited a significantly higher stained area than the TCP control group. Importantly, BGH gave rise to more stained areas than the other three groups after 1 and 2 weeks of culturing under both (−) and differentiation (+) conditions. We can also see that the staining intensity of BG was stronger than that of cell-laden LH, probably due to a combination of bioactive bone mineral release from BG, including Ca^2+^, SiO_4_^4–^, and PO_4_^3–^ ions and its higher compressive strength and Young’s modulus compared to cell-laden LH. Overall, we can conclude from Figure 4b that the ALP-stained area was highest on BGH, reaching 81.66% compared to BG and cell-laden LH that only expressed 36 and 16.66%, respectively. A similar trend was also seen after 2 weeks of culturing.

Similarly, the ALP activity results exhibited higher values in all groups in the presence of differentiation media (+) compared to TCP (Figure 4b). Notably, BGH gave rise to higher ALP activity than the other three groups after 1 and 2 weeks of culturing with both (−) and differentiation (+) culture media. The measured ALP activity was 2-fold higher on BGH than BG and 5.4-fold higher than cell-laden LH in (−) media, respectively. A similar trend was also seen after 2 weeks of culturing. On the other hand, the BGH scaffold resulted in a 7.1-fold higher value than that of cell-laden LH and 3.7-fold higher than the value found on BG in (+) media. The ALP activity of BG was 2.6- and 1.9-fold higher than that of cell-laden LH in complete (−) and differentiation (+) media, respectively. The ALP activity of BG was higher than cell-laden LH, probably due to the release of bioactive bone mineral ions (Ca^2+^, SiO_4_^4–^, and PO_4_^3–^) from BG in combination with its higher mechanical properties compared to cell-laden LH. BGH displayed a more significant ALP upregulation than BG ± and cell-laden LH ±, possibly because of higher compressive strength, compressive modulus, and a more bio-friendly 3D combinatorial environment caused by the release of a more combinatorial biomineral mixture. Notably, we can conclude that the scaffolds, especially BGH, keep their osteogenic properties even without differentiation media.

#### 3.6.4 Alizarin Red S Staining

Matrix mineralization occurs during the maturation of osteoblast cells, and therefore, it is important to examine this hard phase during cell culture as well to get a feeling of the bone healing capacity before moving to the implantation phase. This hard phase is mainly dominated by calcium phosphate granulates. We thus tried to quantify the calcium mineralization level on the different combinations after 2 and 3 weeks of rBMSCs culture by Alizarin Red S (binds to calcium) staining and a commercially available calcium spectroscopic assay (Figure 4c,d). From Figure 4c, we could see reddish dots reminiscent of calcified nodules on both BG and cell-laden LH after 3 weeks in (−) and differentiation (+) culture media. Similarly, we observed that BGH leads to a higher Alizarin red-stained area in both ± culture media than TCP, BG, and LH (Figure 4c). This qualitative trend was confirmed by quantitative Alizarin Red S area analysis in Figure 4d. From here, a similar trend was seen with the highest area found on BGH at 90.33% compared to BG and cell-laden LH at 67.66 and 63%, respectively, in the absence of differentiation media (−). This trend prevailed in differentiation media (+), with the highest area observed on BGH at 95.33% compared to BG and cell-laden LH at 79.33 and 69.33%, respectively.

To further confirm calcium level deposition, a colorimetric assay was performed (Figure 4). From these results, we can see that even after 2 weeks, the calcium deposition on BGH was significantly higher than BG (±) and cell-laden LH (±). Here, after 2 weeks, the calcium deposition was 2.5-fold and 1.6-fold higher than that of BG and cell-laden LH in (−) media, respectively. However, the calcification of the BGH was increased to become 2.5-fold higher than BG and 1.3-fold compared to the cell-laden LH group in (+) media after 2 weeks of cell culture.

Similarly, we observed a higher calcification on BGH than BG and cell-laden LH groups in both (−) and (+) media after 3 weeks of culture. Specifically, the calcium concentration of BGH in (+) media after 3 weeks was 0.53-fold higher compared to BG and cell-laden LH at 0.41 and 0.28, respectively (Figure 4c). Overall, we can thus conclude that BGH facilitated higher calcium mineralization than BG and cell-laden LH after both 2 and 3 weeks of culture, even in differentiation-factor-free media, supporting the conclusions drawn from the ALP results. These quantitative and qualitative assays strongly suggest that BGH supports native-like bone mineralization even without differentiation factors.

#### 3.6.5 Mineralization-Inducing Capacity

Hydroxyapatite (HA) formation is a key indicator of bone mineralization as it is the main component of the hard phase of bone.^88^ Here, we have examined the mineralization-inducing capacity of our scaffolds by immersing them in a SBF solution for 14 days and analyzing them with SEM (Figure 4e). We observed mineral precipitates on the surface of all the three groups (Figure 4e). The SEM image for BGH showed a continuous layer formation consisting of nanogranulates (Figure 4e). The continuous mineral layer formation on the BGH surface differed from what we observed on BG and LH (Figure 4e). For instance, compared to BG, these nanomineral deposits covered almost all of BGH.

On the other hand, the deposits on LH were more uneven, exhibiting large randomly dispersed crests. In addition, the size of the precipitates appeared larger (micron size) than those observed on BGH and BG (nano- to the submicron size). These differences could be caused by the fact that smaller-sized particles can densify more and thus give rise to a smoother mineralized interphase. To follow up on these results and ensure that the mineral deposits were HA, EDX analysis was performed on BG, LH, and BGH. We specifically analyzed the amount of calcium (Ca) and phosphorus (P), which are the main components of HA. EDX of the formed particle deposits on BG and BGH showed that the deposits were primarily composed of phosphorus (P) and calcium (Ca) (Figure 4e). The Si and C peaks can be attributed to bioactive glass and the silicate phase of Laponite, whereas the presence of Ca and P peaks can be attributed to the hypothesized HA formation. The EDX measurements showed that the Ca/P ratios for BG and LH were 2.13 and 2.03, respectively, decreasing to 1.75 on BGH, which is close to the Ca/P ratio of stoichiometric HA, 1.67 (Figure 4e).

To further confirm the speculated HA formation, we examined the mineralized scaffolds using XRD and FTIR (Figure 4f). The XRD patterns of BG, LH, and BGH are shown in Figure 4f. From here, it was found that all samples showed the same diffraction patterns overlapping with that of crystalline HA powder and following the standard JCPDS card no. 01-072-and 1243 °C.^15,89^ We could identify three important HA-related peaks at 2θ values of 26 °C, 31.7 °C, and 32.2° corresponding to the (002), (211), and (112) crystalline planes of HA, respectively (Figure 4f). Notably, the intensity of the diffraction peaks from BGH was highest, which could be related to its higher degree of mineralization per our discussion of the SEM results (Figure 4f). Similar trends are observed from the FTIR spectra (Figure 4f). The peak at 791 cm^–1^, something characteristic of HA and attributed to the bending vibration of carbonate (CO_3_^2–^), was observed for all the three samples (Figure 4f). In addition, peaks at 1087, 610, and 570 cm^–1^ were also identified, corresponding to the stretching and deformation vibrations of phosphate (PO_4_^3–^) groups.^90^ Therefore, the FTIR analysis further supported the presence of HA minerals in these three groups. Importantly, the stronger peaks observed from BGH were in line with SEM, EDX, and XRD results. Overall, it can be concluded that the mineral phase observed on BGH after 2 weeks in SBF was closer to the inorganic composition of natural human bone than the other groups.

We believe that the bioactivity of BG is very high and significant due to its ability to promote HA formation. This can be attributed to its bone-like constituents, including Ca, P, and Si, which all have a positive role in the rapid exchange of ions that occurs upon the dissolution of bioactive glasses leading to HA formation.^91^ A less pronounced HA formation was observed on LH, albeit still there. For this reason, the combination of the two into a single system could, in theory, support a higher biomineralization degree—exactly as we observed. This is most likely also due to a higher available surface area on BGH arising from both the porous structure of BG and LH compared to the individual units alone. Another important factor is the increase in the number of mineral types released from both BG and LH, giving rise to more combinatorial and native-like mineral reservoirs.

### 3.7 Histological Assessment of Bone Formation in Critical-Sized Rat Calvarial Defects

**Figure 5:**
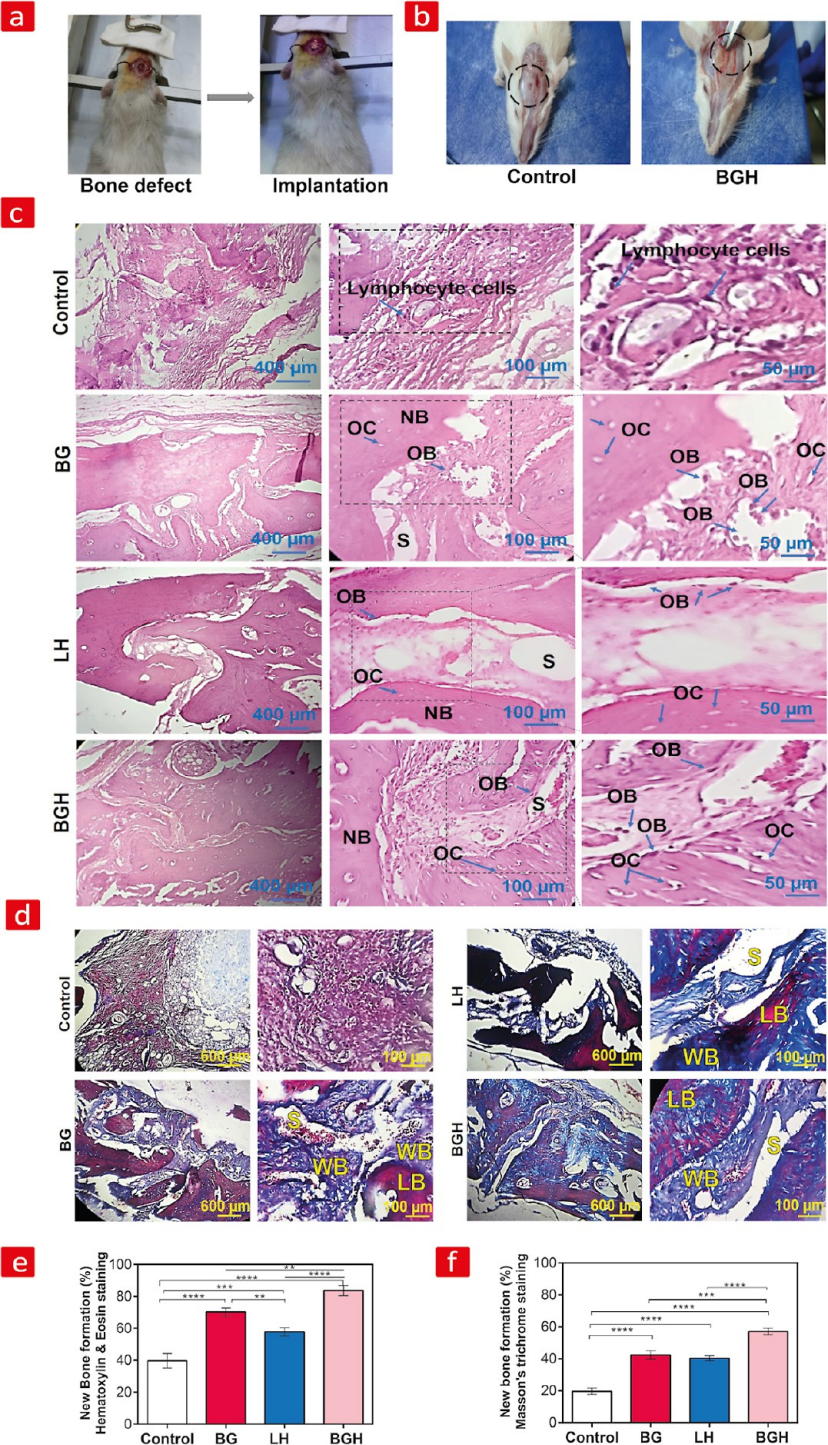
In vivo studies. (a) Surgical procedure employed for creating a rat calvarial bone defect model and the implantation of our scaffolds in the defect area. (b) Representative images of bone regeneration in control and BGH after 8 weeks post-surgery. The bone defects in the control group were left empty without any treatment. The circles show the regenerated bone tissues. Histological evaluation of bone healing after 8 weeks is shown in (c) via H&E stained images of all groups. New bone formation (NB), scaffold (S), osteoblast (OB), and osteocyte (OC) cells have been highlighted here. (e) Masson’s trichrome (MT) histological staining showing woven bone (WB) and lamellae of bone structures (LB). (d,f) The new bone formation observed from H&E and Masson’s trichrome staining was quantified through image analysis and presented here, respectively. **p < 0.05, ***p < 0.001, and ****p < 0.0001: statistically significant differences.

We used a critical-sized rat calvarial bone defect for our in vivo experimental model (Figure 5a). The defects were filled with cell-free BG, LH, and BGH implants, while the control group was kept empty. After 8 weeks of implantation, the skin on the head of the rat skull was gently removed, and the bone healing degree of empty defect, BG, LH, and BGH, was examined with histology (Figure 5b). The circles in the images show the regenerated bone tissues. We could observe that the defect area in the control group was not filled out, indicating that the bone defect could not regenerate itself following its critical-sized nature (Figure 5b). Importantly, there are no traces of holes in the BGH group, and the defect area was almost filled with new tissues.

Moreover, most of the scaffold was replaced with new bone tissues, meaning that BGH perhaps could lead to a near-perfect bone healing (Figure 5b). To confirm these observations, histological analysis was performed. In this regard, we used Hematoxylin & Eosin (H&E) and Masson’s Trichrome (MT) stainings to assess this in more depth for all groups (Figure 5c–f). H&E mainly stains the cell nuclei (dark purple) and the bone matrix (pink), while MT colors collagen blue and mature bone red. MT staining is more detailed than H&E as it can be used to distinguish immature collagenous bone (sparse in mineralization and woven-like) with more dense lamellae bone via a blue and red color, respectively.

In terms of H&E, the degree of new bone formation for cell-free BG, LH, and BGH groups is evident from Figure 5c, whereas we could only see fibrous tissue formation with small quantities of new bone and lots of lymphocyte cells in the empty defect. Regarding BGH, the new bone filled almost the entire critical-sized defect area, as characterized by the pink color of eosin, whereas less bone formation and immature regenerated bone tissue were observed in the BG and LH groups, compared to the BGH group, suggesting that all groups except the control group could give rise to measurable bone healing. Importantly, high-resolution images of the H&E-stained sections demonstrated the presence of native osteoblasts (OB) and osteocytes (OC) distributed throughout the bone tissues, which most likely are there because of native bone progenitor cells that have migrated into the implants from the surrounding tissue. These can then differentiate into osteoblast cells and, over time, mature into osteocytes. Overall, the H&E staining analysis confirmed that BGH gave rise to the highest bone volume fraction, reaching about 83.66%, whereas LH and BG gave rise to only 57.66 and 70.33% bone formation, respectively (Figure 5e).

MT was used to obtain a more detailed picture of the structure of the regenerated bone tissue (Figure 5d). From Figure 5d, we could see the presence of woven-like bone (WB) and a lamellae-shaped bone matrix (LB) in all groups (Figure 5d). It is important to note that when a native bone is regenerated too rapidly, collagen fibers form in randomly oriented bundles without preferred organization. This type of bone, unlike lamellar bone, is called woven bone and is characterized by irregular calcification and disordered collagen fiber bundles. There were no signs of such bone structures in the control group. However, we observed more pronounced LB formation in BGH than in the other groups. Overall, we could conclude from the MT staining that more mature bone tissue and a higher amount of LB were observed in BGH than the other groups, suggesting a more native-like bone formation.

Interestingly, there was almost no remaining scaffold (S) material left in the BGH group compared to BG and LH (Figure 5d,f). Moreover, the results of quantitative MT analysis confirmed the visual assessments with the highest amount of new bone found in BGH, reaching about 57%, while LH and BG only gave 40.33 and 42.33%, respectively (Figure 5f). Thus, the in vivo results align with the in vitro results discussed above. It seems like a bone remodeling process was activated in the cell-free BG, LH, and BGH groups due to the presence of both OBs and OCs within the newly formed bone. This could be due to the migration of native bone progenitor cells that, over time, could give rise to mature bone.

#### 3.7.1 In Vivo Osteogenic Activity

**Figure 6:**
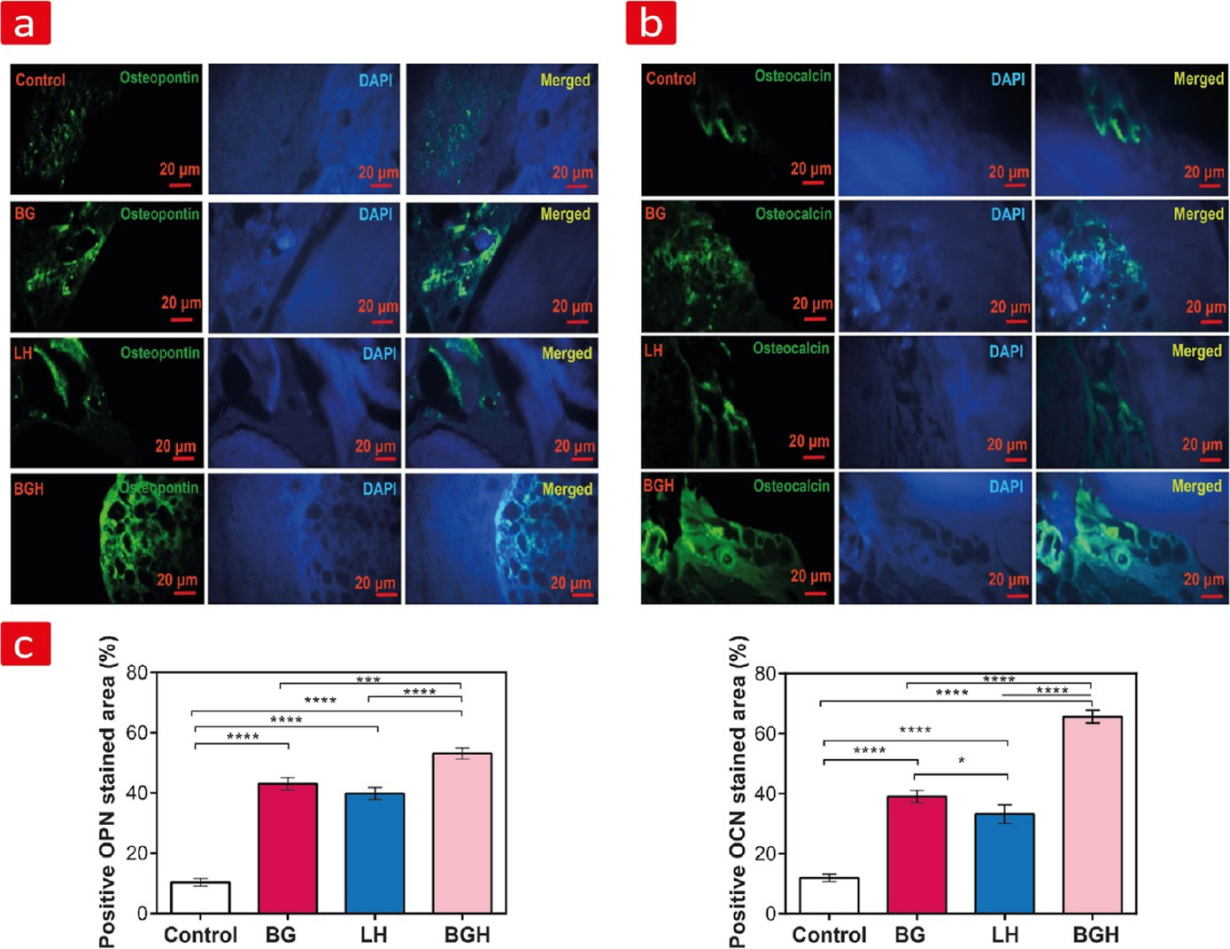
Immunohistochemical OPN and OCN staining. (a) OPN and (b) OCN detected in the newly formed bone. The green-stained area indicates the expression of OPN and OCN, and the blue staining corresponds to the surrounding tissue. (c) Expression levels of OPN and OCN as measured by ImageJ analysis. *p < 0.05, ***p < 0.001, and ****p < 0.0001: statistically significant differences.

In vivo osteogenesis was further assessed by immunohistochemistry staining of OCN and OPN for each of the cell-free groups after 8 weeks. The expression of these mid-range bone biomarkers is clearly visible in Figure 6a,b, and the quantitative analysis of their positive stained area is shown in Figure 6c. The expression of OCN and OPN proteins was detected in appreciable amounts in all groups, except for the control group, which was almost below the detection level. Indeed, we could observe stained areas corresponding to OPN (51.3% ± 2) and OCN (65.66% ± 2.08) on BGH, while BG and LH gave [OPN (43.1% ± 1.8) and OCN (39.03% ± 1.08)] and [OPN (39.86% ± 1.6) and OCN (33.2% ± 2.04)], respectively. This high protein expression seen from BGH further confirmed that the osteogenic activity of BGH was better than that of BG, LH, and control groups, something which further supported the hitherto discussed upregulated osteogenesis on BGH compared to all other groups. What is more, the expression levels of OPN and OCN proteins were lower in the cell-free LH groups in comparison to the BG (*p < 0.05) and SH (****p < 0.0001) groups. All these results indicated that the BGH scaffold is better at promoting osteogenic differentiation of native cells toward bone formation and can thus be considered as a more favorable environment for expression of osteogenic ECM in vivo.

## 4 Discussion

Bottom-up tissue engineering is a promising approach to create biomimetic structures that replicate the hierarchy and biofunctionality of native human tissues. Bottom-up assembly of living blocks enables the creation of multi-cellular-rich structures or multi-component cell-biomaterial synergies, which have the potential to develop more robust and functional humanized tissues for therapies and disease models. Compared to top-down approaches, which involve cell seeding in supporting porous 3D scaffolds, bottom-up approaches are better at replicating the unit-repetitive modular design found in native human tissues. Nanoparticles such as nanosilicates have the potential to incorporate bioinstructive cues into bottom-up bioarchitectures to promote 3D maturation and improve biofunctionality. Nanoparticles can be incorporated to provide bioinstructive cues and spatiotemporally control biophysical signals presentation to cellular building blocks, such as generating bioinstructive gradients of different growth factors for promoting endothelial sprouting or establishing osteochondral interfaces. Laponite nanoparticles are being widely studied in 3D nano-bioprinting because of their natural bioactivity, mechanical strength, and ability to sustain the delivery of bioinstructive signals. Notably, the combination of cell-rich constructs with biomaterials at multiple levels, from nano to macro, is a promising bottom-up approach to recreate the structure, organization, and physiology of native tissues in 3D constructs. Achieving a self-regulated response to biomolecular cues is crucial for the 3D microtissues to perform their intended functions. Creating implantable multi-scale assemblies that mimic human tissue architecture and respond to the microenvironment is complex, but essential for successful integration into host tissues.^92^ Hydrogels and scaffolds have been used separately to create native bone tissues. However, they both suffer from limitations. Hydrogels have poor mechanical strength, making them fail easily under heavy loads, and ceramic scaffolds do not provide a sufficient native-like environment for cells to perform optimally. On their own, they cannot facilitate sufficient bone formation. Indeed, after 8 weeks, the range has hitherto been 5–60%,^20,93−98^ and for cell-free scaffolds, 2–42% after 8 weeks,^99−107^ respectively. In this study, we have tried to overcome these limitations by using a fairly new approach in the field compensating for both the soft nature of the bone ECM and the hard mineral-like properties of mineralized bone. Specifically, we have combined nanosilicate-reinforced alginate with a BG scaffold. A formulation that we theorized could enable a tradeoff to sustain their respective benefits. Our design is both multi-leveled (scaffold and hydrogel) and hierarchical, covering geometrical features ranging from nanometer to micrometer, exactly like native bone (Figure 1). Notably, the mineral composition of our combination system (BGH) was supercombinatorial, with our BG being made from Ca, Si, and P and the soft phase from Si, Mg, Li, and Na. This unique combinatorial approach proved superb compared to previously tested implants in calvarial defects and yielded almost near-perfect bone healing.

To assess the bioactivity of the engineered combination scaffolds, we studied the release of minerals from both BG and BGH and found a substantial and sustained release of important mineralites capable of maintaining proper bone functioning, including Ca^2+^, SiO_4_^4–^, PO_4_^3–^, Mg^2+^, and Li^+^ ions. For this reason, BGH is very combinatorial as it can release a complex mixture of ions from both the hard BG phase and from the softer LH phase. This is indeed a remarkable result, as several studies have hypothesized that releasing these minerals, especially from Laponite, plays a significant role in cellular activities and can promote osteogenesis in the absence of differentiation factors.^45,46^ Furthermore, Young’s modulus of BGH was 1.22 and 10.4 times greater than that of BG and LH in dry conditions. We believe that this increase in mechanical properties can be attributed to a decrease in pores and porosities compared to BG and LH, higher material density, as well as a stiffer structure stemming from the formation of hydrogen bonds between silicate groups in BGH and the interactions of carboxylic groups in alginate with silica groups of Laponite. Overall, BGH was thus more native-like in its mechanical properties and chemical structure.

In vitro osteogenic activity was then assessed by immunocytochemistry staining and looking at the expression of important osteogenic genes. Here, we noticed that both the secretion of the mid-range markers—OPN and OCN—and their gene expression were more pronounced than BG and cell-laden LH. We believe that this high expression of osteogenic protein markers may be attributed to the ions released from BG, including Ca, P, and Si, and the release of Laponite mineral constituents including Si, Li, and Mg, which all have a positive role in the osteogenic differentiation according to the recent literature.^45,49,51,108,109^ Additionally, we evaluated the osteogenic capacity of all combinations by looking at ALP expression and activity. It was found that BGH showed significantly more ALP positively stained areas and resulted in higher ALP activity compared to the other groups in both (±) differentiation media after 1 and 2 weeks. The same trend beholds regarding in vitro bone mineralization for all time points. The outstanding performance of BGH is likely due the release of its combinatorial mineral constituents to the other groups. The observed higher differentiation values for BGH than BG and LH could be attributed to the higher observed release rate of bioactive ions (Figure 2f) into culture media from BGH compared to BG and LH (Figure 2f). To this end, recent studies have shown that different nanosilicate plates can play an important role in bone differentiation in a growth-factor-free environment.^45,46,51,110,111^ This has been speculated to be due to the absorption of the clay particles inside cells and their intracellular degradation into mineral products, including lithium, silicate, and magnesium—minerals considered essential elements involved in bone formation.^45,112^ These facts about in combination with BGH more native-like mechanical properties compared to the others could all together explain its amazing osteogenic properties.

Additionally, histological assessment of in vivo bone formation in a critical-sized rat calvarial defect was carried out with cell-free BG, cell-laden LH, and BGH-based implants after 8 weeks. These results confirmed that the defect area in the BGH group was almost filled with new bone tissues, resulting in an almost near-perfect bone healing (about 83.66%). It should be noted that if the root cause of a bone defect, such as infection or trauma, is not properly addressed, a relapse may occur. The regenerated bone may not be as robust as the original bone, leaving it vulnerable to fractures or other types of damage.^113^ However, our research has demonstrated that implanting nanosilicate scaffolds into rat calvarial defects resulted in a significant 84% increase in new bone formation after 8 weeks of healing, without any relapse. The new bone was also well integrated with the surrounding tissue and exhibited similar mechanical properties to natural bone. Our bone defect model healed entirely and remained stable over the long term. Also, H&E staining confirmed the osteogenic differentiation of native cells from the surrounding tissue into osteoblasts (OB) and osteocytes (OC). These results are remarkably better than the few studies on multi-leveled scaffolds like this one.^63,67−70^ For instance, a recent study examined the in vivo performance of biphasic calcium phosphate/hyaluronic acid-gelatin in a rabbit femur defect.^69^ Compared to our study, these authors reported much lower bone formation (30%) using micro-CT analysis after 2 months of implantation^69^ Another study evaluated bone regeneration of cell-free poly-l-lactide-*co*-trimethylene carbonate scaffolds combined with modified human platelet lysate hydrogels implanted in rat calvarial defects. The results revealed that about 15% bone regeneration was obtained after 2 months of implantation by using in vivo CT-scanning technique.^70^ Even after 3 months, they could not achieve the same bone formation as observed herein, with the increase being around 43.2% only.

Taken together, our study suggests that incorporating Laponite (hydrogel) into a hard scaffold can support in vitro production of mineralized bone-like tissue in a differentiation-factor-free environment and unprecedented in vivo bone regeneration. We believe that the inclusion of bone progenitor cells in our systems could perhaps facilitate a complete bone repair in a shorter time. The inclusion of collagen fibers and osteogenic proteins such as osteopontin and osteocalcin could also make the system even more native-like and increase the bone formation rate as well.^114−117^ The combination of encapsulated cells and collagen fibers could herald an implant capable of facilitating a near-perfect bone healing even after 4 weeks. We have indeed leaped forward from traditional non-native bone implants to biomimetic implants, with a higher osteogenic potential than what is usually reported in the field. Indeed, this methodology could potentially create a new paradigm in bone tissue engineering. Also, such scaffolds can be combined with nanoelectronics and stimuli-responsive materials to yield smart materials that can wirelessly monitor and automatically detect and track bone defects, respond to various stimuli, and, if needed, automatically repair the injuries. Recent studies have already combined nanomaterials and cell-laden hydrogels to reveal new promising cyborg-like systems.^51,116^ Nanosilicates have the potential to improve the osseointegration of orthopedic implants and stimulate bone growth, making them a promising option for targeted drug delivery and regenerating bone tissue. Their unique properties, such as biocompatibility, high surface area, and osteoconductivity, make them well suited for clinical applications. Additionally, they can be easily tailored for specific clinical purposes, which makes them a cost-effective option. Although nanosilicate implants have shown low toxicity and biocompatibility in vitro and in vivo studies, there are still challenges that need to be addressed before they can be used in clinics. More research is required to understand their long-term effects on the human body, and regulatory approval and further development will be necessary for their successful translation into clinical practice and commercialization.^84,118^

Nanosilicate implants have displayed potential for therapeutic use in bone tissue regeneration; nevertheless, potential risks associated with their implementation should be carefully taken into account. The potential for immunological reactions to the nanosilicate material presents a potential risk, as it may cause inflammation or other negative consequences. Additionally, unexpected long-term effects may be observed and are not yet fully understood. Among the primary concerns associated with nanosilicate implants is the possibility of toxicity, despite research suggesting their safety. Poor design or administration of the implant may lead to adverse effects such as organ damage, inflammation, or allergic reactions. Further, the body may potentially reject the implant even though in vitro and animal studies indicate biocompatibility. Hence, the risks and benefits of nanosilicate implants should be comprehensively evaluated, with continuous monitoring of safety and effectiveness in clinical settings.^119,120^

## 5 Conclusions

We have successfully utilized a multi-level scaffold for bone tissue engineering applications. Our composite consisted of a soft nanosilicate phase and a harder mineral-based phase made from BG. The combination of the two captured the hierarchical structure of bone quite well while simultaneously giving rise to the release of a combinatorial mixture of ions including Li, Mg, Si, Ca, and P. Notably, we demonstrated that the native-like mineral composition could turn progenitor cells into mature bone cells in a differentiation-factor-free environment. This observation also prevailed in vivo. Indeed, we could achieve a record-high bone healing within a rat calvarial defect after only 8 weeks of culture. Such near-perfect bone healing is rarely seen in the field and might herald a new era in repairing musculoskeletal defects. We speculate that this is intimately linked with the migration of native progenitor cells into the softer 3D phase of the implant. Here, they can be guided by the nanosilicate phase into mature osteoblasts capable of secreting a mineralized matrix. Importantly, we also observed osteocytes, which most likely stem from further differentiation of the osteoblast cells, indicating a complete bone healing cycle. Future studies could coat the nanoplates with chemoattractants, such as stromal cell-derived factor 1 (SDF-1) and bone morphogenetic protein 2 (BMP-2), to further increase the migration of cells into the implant. In combination with bioactive molecules such as collagen, osteopontin, or osteocalcin, we might even reach higher bone formation rates in vivo. A limitation of our study was the unavailability of micro-CT. Thus, future studies will include micro-CT characterization of scaffolds to evaluate the new bone formation and study bone volume, density, and microarchitecture.

## Supporting Information Available

Concentration release studies of Na^+^ from BGH; rheological studies of LA and LH; in vitro cell studies on BG, LH, and BGH scaffolds; cell viability studies on BG, LH, and BGH scaffolds; and expression of the osteogenic-related gene (PDF)

## Notes

The raw/processed data required to reproduce these findings cannot be shared at this time due to technical or time limitations.
