# Supplementary material for: Multi-leveled Nanosilicate Implants Can Facilitate Near-Perfect Bone Healing
Source: ACS Appl Mater Interfaces. 2023 Apr 19;15(17):21476–95. doi: 10.1021/acsami.3c01717 (PMC10165608; doi:10.1021/acsami.3c01717)
Supplement: Supplementary file 1 — am3c01717_si_001.pdf [file am3c01717_si_001.pdf]

Supporting Information

Multi-leveled Nanosilicate Implants Can Facilitate Near-Perfect Bone Healing

Mozhgan Keshavarz [a,b], *Parvin Alizadeh* [a,*], *Firoz Babu Kadumudi* [c], *Gorka Orive* [b,d,e,f,*], *Akhilesh K. Gaharwar* [g], *Miguel Castilho* [h,i,j], *Nasim Golafshan* [j], *and Alireza Dolatshahi-Pirouz* [c,*]

[a] Department of Materials Science and Engineering, Faculty of Engineering & Technology, Tarbiat Modares University, P. O. Box: 14115-143, Tehran, Iran

[b] NanoBioCel Research Group, School of Pharmacy, University of the Basque Country (UPV/EHU), Vitoria-Gasteiz 01006, Spain

[c] Technical University of Denmark, DTU Health Tech, Center for Intestinal Absorption and Transport of Biopharmaceuticals, 2800 Kgs. Lyngby, Denmark

[d] Biomedical Research Networking Centre in Bioengineering, Biomaterials and Nanomedicine (CIBER-BBN), Vitoria-Gasteiz 01006, Spain

[e] University Institute for Regenerative Medicine and Oral Implantology - UIRMI (UPV/EHU-Fundación Eduardo Anitua), Vitoria-Gasteiz 01006, Spain.

[f] Bioaraba, NanoBioCel Research Group, Vitoria-Gasteiz 01006, Spain

[g] Department of Biomedical Engineering, College of Engineering, Texas A&M University, College Station, TX 77843, Texas, USA

[h] Department of Biomedical Engineering, Eindhoven University of Technology, Eindhoven 5612 AE, The Netherlands

[i] Institute for Complex Molecular Systems, Eindhoven University of Technology, Eindhoven 5612 AE, The Netherlands

[j] Department of Orthopedics, University Medical Center Utrecht, Utrecht University, Utrecht 3508 GA, The Netherlands

*Corresponding Author.

E-mail addresses: P-Alizadeh@modares.ac.ir (P. Alizadeh), gorka.orive@ehu.es (G. Orive), aldo@dtu.dk (A. Dolatshahi-Pirouz)

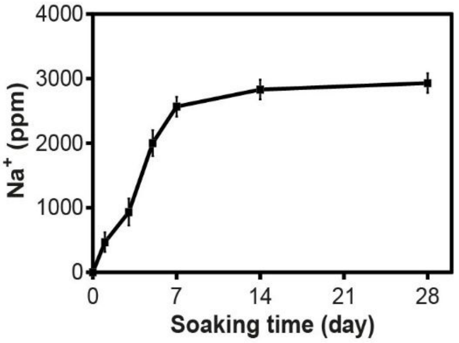

Figure S1. The concentration of released Na^+ from BGH over 28 days is shown here.

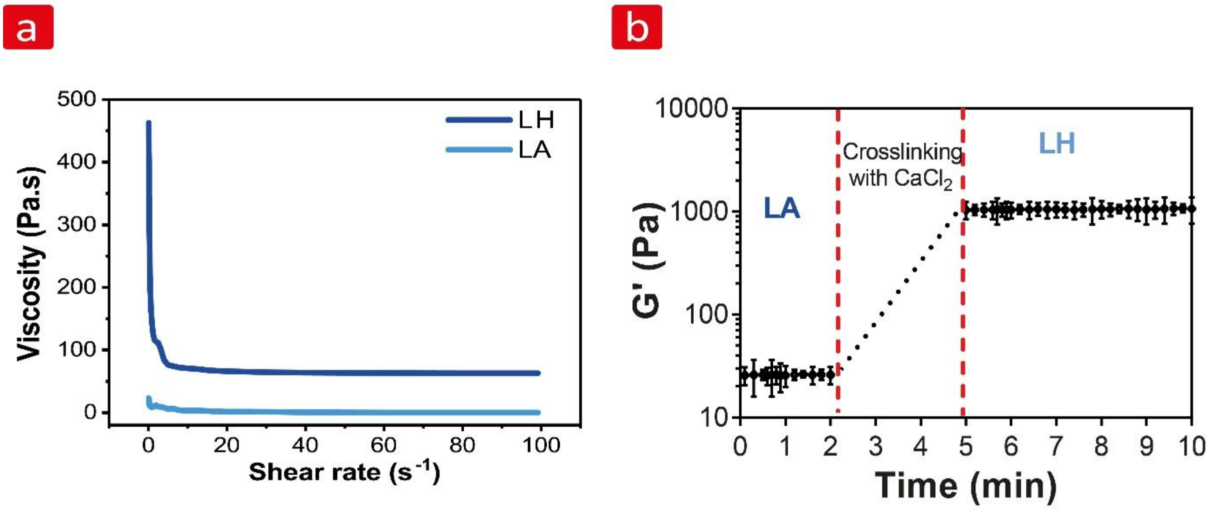

Figure S2. Rheological studies. (a) Viscosity of LA and LH as a function of shear rate at room temperature. (b) Storage modulus of LH before and after the ionic crosslinking process.

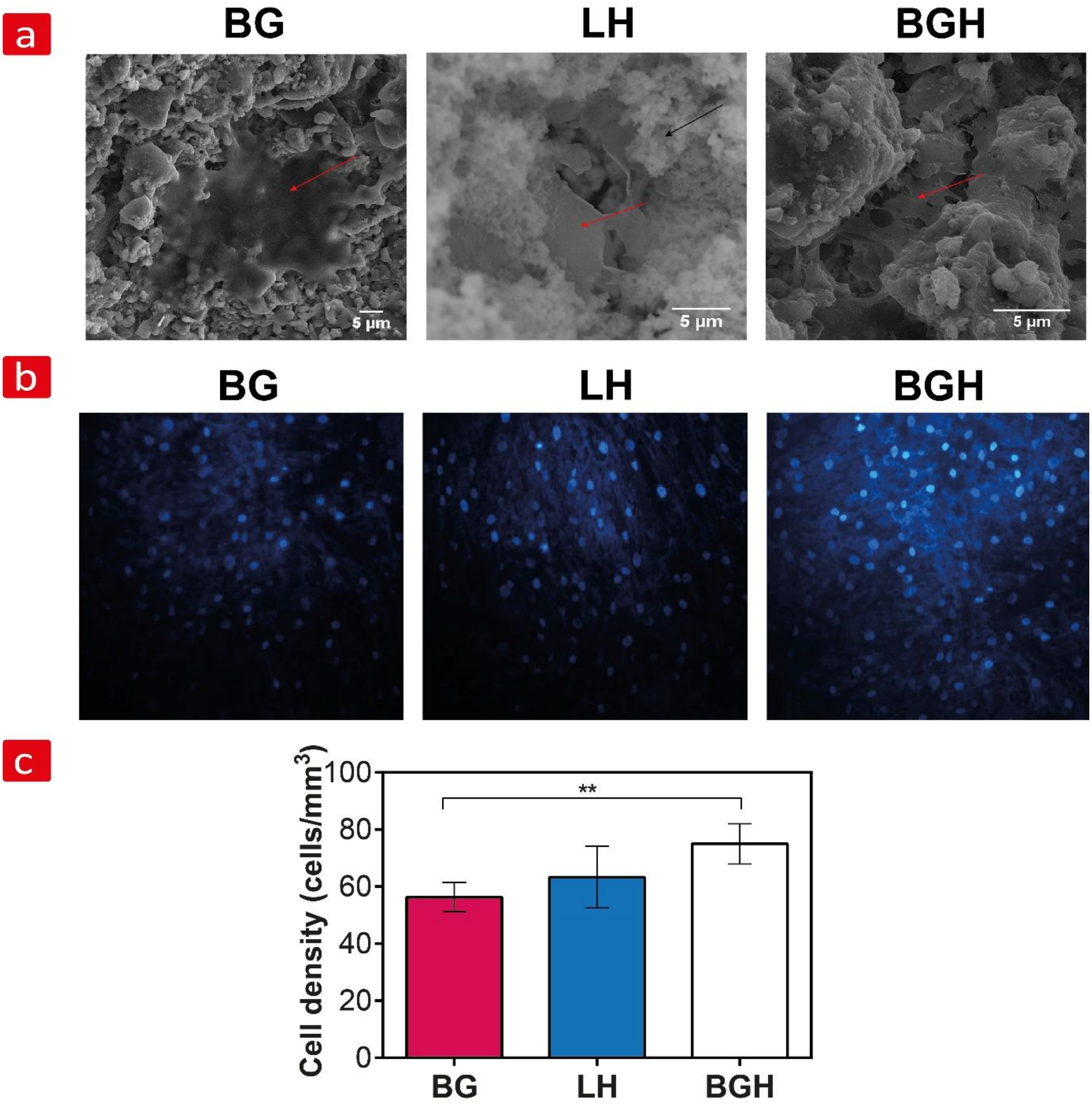

Figure S3. In vitro cell studies on BG, LH, and BGH scaffolds. (a) SEM images depicting cell attachment and spreading on BG, LH, and BGH after 7 days of culture. (b) Nuclei (blue) staining of rBMSCs within BG, LH, and BGH scaffolds, respectively, at day 14. (c) Cell density was calculated through rBMSCs nuclei staining with DAPI at day 14, scale bars = 200 μm. **p < 0.001: statistically significant differences.

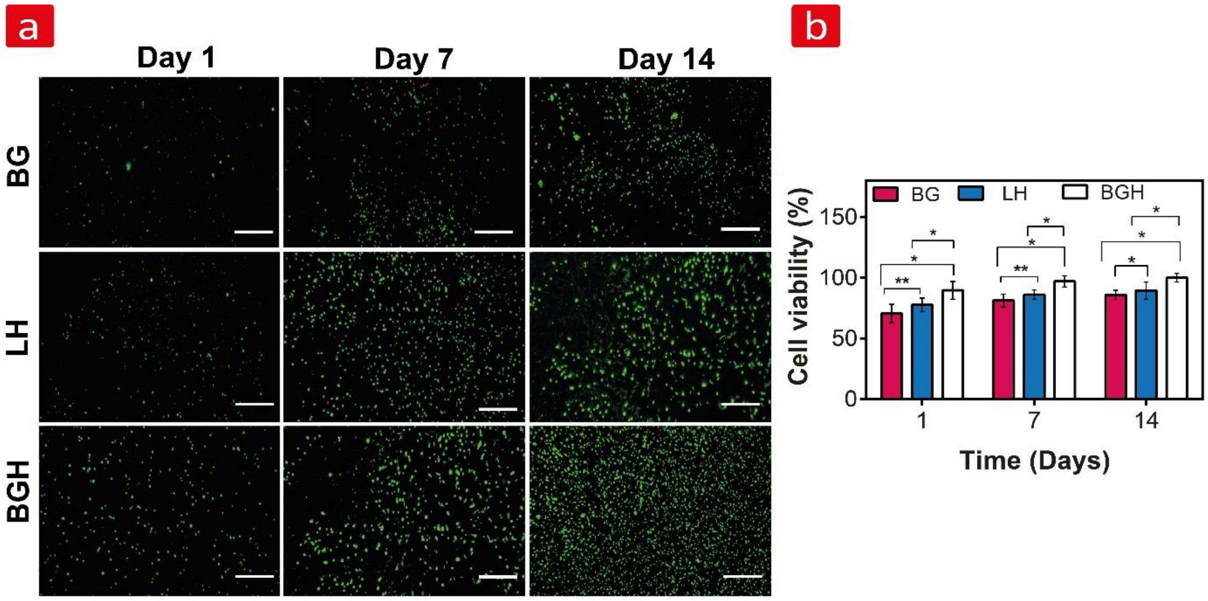

Figure S4. In vitro cell studies on BG, LH, and BGH scaffolds. (a) Cell viability: (live cells: green, dead cells: red) staining of rBMSCs seeded on BG and BGH and encapsulated in LH after day 1, 7, and 14, scale bars = 200 µm. (b) Cell viability of rBMSCs quantified from the above confocal images, scale bars = 200 µm. *p < 0.05 and **p < 0.001: statistically significant differences.

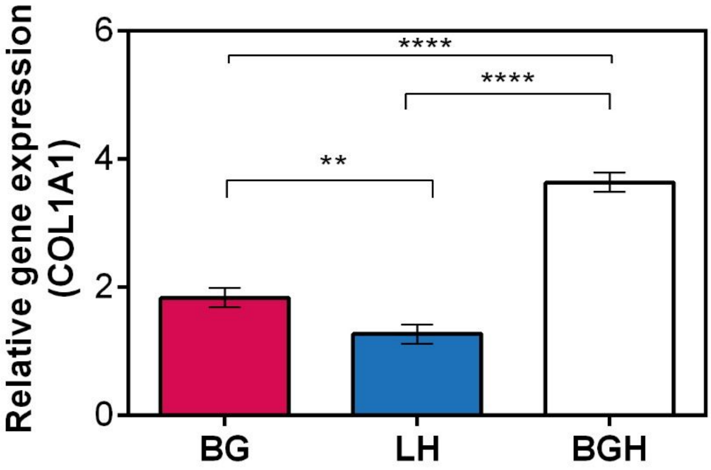

Figure S5. Expression of the osteogenic-related gene. COL1A1 after 14 days of osteogenic induction. (One-way ANOVA was used; **, p < 0.05; ****, p < 0.0001).
